# Transition Metals Coordination by Bis-imidazole-calix[4]arene Ligands with and Without Pyrene Units Grafted at the Large Rim

**DOI:** 10.3390/ijms252011314

**Published:** 2024-10-21

**Authors:** Ivana Nikšić-Franjić, Dijana Pavlović Saftić, Vilko Smrečki, Benoit Colasson, Olivia Reinaud, Ivo Piantanida, Aleksandar Višnjevac

**Affiliations:** 1 Laboratory for Chemical and Biological Crystallography, Division of Physical Chemistry, Ruđer Bošković Institute, Bijenička Cesta 54, 10000 Zagreb, Croatia; ivana.niksic-franjic@irb.hr; 2 Laboratory for Biomolecular Interactions and Spectroscopy, Division of Organic Chemistry and Biochemistry, Ruđer Bošković Institute, Bijenička Cesta 54, 10000 Zagreb, Croatia; dijana.pavlovic.saftic@irb.hr; 3 NMR Center, Ruđer Bošković Institute, Bijenička Cesta 54, 10000 Zagreb, Croatia; smrecki@irb.hr; 4 Laboratoire de Chimie et de Biochimie Pharmacologiques et Toxicologiques, Université Paris Cité, CNRS, F-75006 Paris, France; benoit.colasson@parisdescartes.fr (B.C.); olivia.reinaud@parisdescartes.fr (O.R.)

**Keywords:** calix[4]arene, pyrene excimer/exciplex, metal coordination, X-ray structures, fluorescence, dehydrogenative coupling

## Abstract

Herein, the presented results show that previously studied DNA/RNA-interacting bis-imidazole-calix[4]arene systems can, in aqueous solutions, efficiently bind a series of biorelevant transition metal cations by coordination with the two imidazole arms at the small rim of their macrocyclic basket. The SCXRD and NMR results structurally characterised the complexes formed by referent bis-imidazole-calix[4]arene with Cu^2+^ and Zn^2+^. In solid-state (crystal), the bis-anilino derivative/Cu^2+^ complex, only upon exposure to the air, undergoes intramolecular dehydrogenative coupling of two neighbouring aniline units, yielding an azo bridge at the large rim of the calix[4]arene basket. In the biorelevant aqueous solution, the comparison of fluorometric titrations of referent calix[4]arene, with its analogues having one or two pyrene units grafted at the opposite (large) rim, revealed moderate-to-strong affinity towards transition metal cations, and, more importantly, a strong impact of pyrene on the binding affinity towards some cations. The pyrene arm(s) significantly diminished the affinity of the calix[4]arene-imidazole ligand towards Cu^+^ and strongly increased the affinity towards divalent Co^2+^ and Cd^2+^ cations. Moreover, the fluorometric response of some studied derivatives was strappingly sensitive to cation type. Since the counter-anion plays only a marginal role, such a change in selectivity is attributed to the intramolecular interaction of pyrene(s) with the calix[4]arene-imidazole system, sterically controlling the metal cation binding site.

## 1. Introduction

Metal cations, many of which are ubiquitously present in living organisms, are not to be overlooked in the context of the interaction of the low molecular weight (LMW) organic ligands and biomacromolecules, since their distinctive electronic, chemical, and photophysical properties often have a decisive impact on the recognition process [1,2]. This impact is very often realized through the coordination of the metal ion by an organic ligand (which is, at the same time, a recognition agent) [3]. Bearing an intrinsic charge, metal ions change the overall charge of the organic ligand, which can decisively affect its capability to bind to a macromolecular chain [4,5]. Many metal ions are also characterised by redox potential and/or a specific level of Lewis acidity, which gives them the additional ability to influence/catalyse bio-essential redox and/or hydrolytic processes in their proximity [6]. Furthermore, the coordination of a metal induces conformational modifications of a parent LMW ligand, rendering it more or less prone to a specific recognition event. For all of these reasons, those small molecules which are, at the same time, likely to recognize specific portions of the macromolecular chain, and coordinate various metal ions, are potentially more powerful macromolecular binders of a greatly enhanced versatility than the organic molecules without a significant metal coordination ability [7].

While metals are normally present in living cells and many of them are essential to support various life processes, they can present an extreme hazard for the biosphere, especially in high concentrations [8]. Many metals, such as cadmium, cobalt, chromium, copper, lead, or zinc, are important contaminants in industrial discharge waters. It is, therefore, critical to develop simple and efficient tools to monitor these concentrations, for obvious environmental and medical applications. Commonly applied analytical techniques like inductively coupled plasma-mass spectrometry or atomic emission spectrometry usually require expensive instrumentation as well as a demanding sample preparation [9]. Unlike these traditional methods, fluorescence spectroscopy is an operationally simple yet sensitive selective and low-cost method to monitor the metal cation concentrations [10]. It does, however, require a design and synthesis of fluorescent chemosensors, molecules capable of entrapping a metal cation and reporting by fluorescence modification [11,12,13,14,15]. The typical design of such chemosensors is based on macrocycles with grafted fluorescent units. For instance, J. Yoon and co-workers have monitored a process they called “molecular taekwondo” by a fluorescence variation on pyrene-armed calix[4]aza crowns in ethanol. For calixarene derivatives bearing simultaneously a crown and an aza-crown ether, the metal ion was found to selectively choose a better-suited binding pocket between these two multidentate ligands grafted to the calixarene core. The processes between Ag^+^–K^+^, Cu^2+^–K^+^, and Ag^+^–Cs^+^ pairs were easily monitored via fluorescence change [16]. These functionalized calixarenes were also investigated computationally by employing the DFT and TD-DFT methods. The obtained results have shown that metal-π and metal-ion repulsive interactions are the main driving forces of the coordination while the influence of the solvent is also significant [17]. L. Yu and co-workers [18] synthesized a highly selective fluorescent chemosensor based on a bis-pyrene calix[4]arene scaffold and proposed a model for fluorescence change upon the addition of Na^+^ [19]. Nam et al. noticed that some ions induce photo-switchable turn on and off fluorescence of a 1,3-alternate calix[4]arene chemosensor containing urea and pyrene moieties. More precisely, the binding of Pb^2+^ between urea subunits results in fluorescence quenching, while the addition of F^−^ turns the fluorescence on [20].

**Scheme 1:**
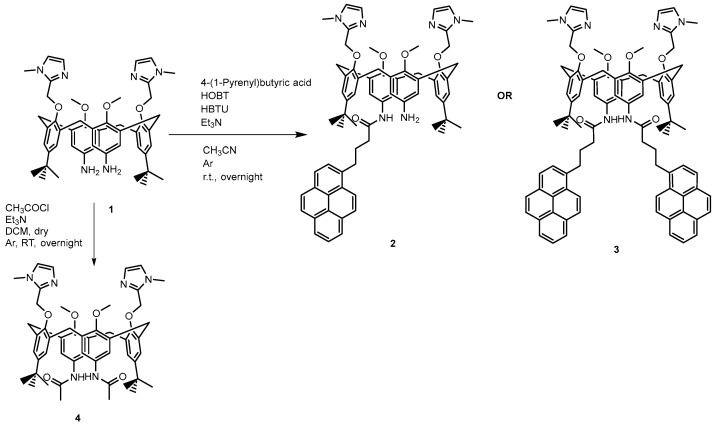
Synthesis of compounds **2**, **3,** and **4** from the parent compound **1**.

We have previously developed a series of both organic- and water-soluble calix[4]arene-based biomimetic systems [21,22,23]. We have shown, thereafter, as a part of our continuous investigation on calixarene-based derivatives for the recognition of a variety of DNA/RNA chains, that some of these edifices may act as powerful binders for polynucleotide chains but only if they bear a permanent positive charge [5]. This finding has, unfortunately, eliminated a large portion of our library of potential binders from further consideration, unless they are chemically converted to the positively charged species. Instead of going through the trouble of lengthy and demanding synthetic modifications, we opted to explore more closely their metal coordination abilities in order to add the desired permanent charge, and we are reporting the first findings of these new studies on our calix[4]arene systems **1** and **4** (Scheme 1). Importantly, our previous studies have shown a remarkable affinity of the analogous calix[4]arene systems to bind monovalent cations like Cu(I) [21]. More recently, we reported on the synthesis of the series of pyrene-armed bis-imidazole calix[4]arene systems and their notable affinity towards both mono- and polynucleotides [24]. Intrigued by our findings, and because of the generally observed ability of pyrene-functionalized calixarenes to act as selective chemosensors for metals, and hence by the possible consequences of the metal binding to the spectroscopic behaviour of our systems, so crucial in monitoring their DNA/RNA binding, we have undertaken a parallel study of the capability of these pyrene-armed bis-imidazole calix[4]arenes (**2** and **3**, Scheme 1) to coordinate various metal cations (including divalent cations), the results of which are also reported here.

## 2. Results

### 2.1. Molecular and Crystal Structures

**Scheme 2:**
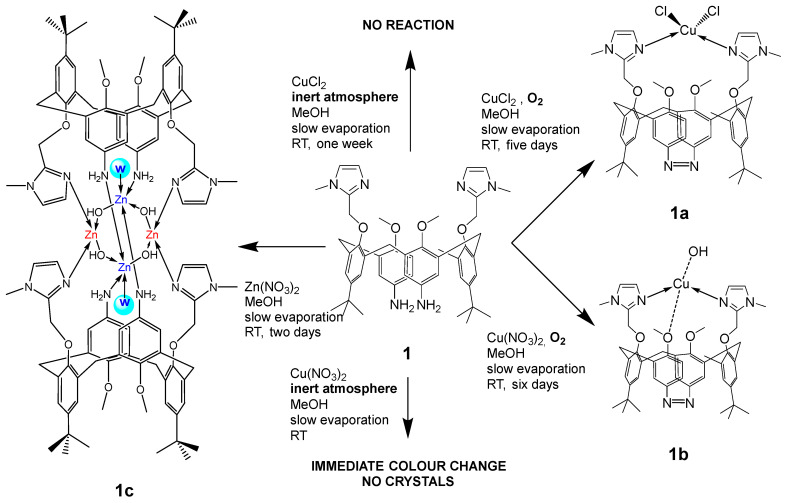
Preparation and crystallization of complexes **1a**, **1b,** and **1c**. In the diagram of **1c,** blue bubbled “w” stands for coordinated water molecules. Blue marked Zn is penta-coordinated, red-marked is tetra-coordinated.

In order to obtain a clearer insight into the modes and sites of interaction of metal cations with the calix[4]arene-based DNA/RNA binders, we have prepared and crystallized series of metal complexes of parent compound **1** and determined their single crystal X-ray structures (Scheme 2).

**Figure 1:**
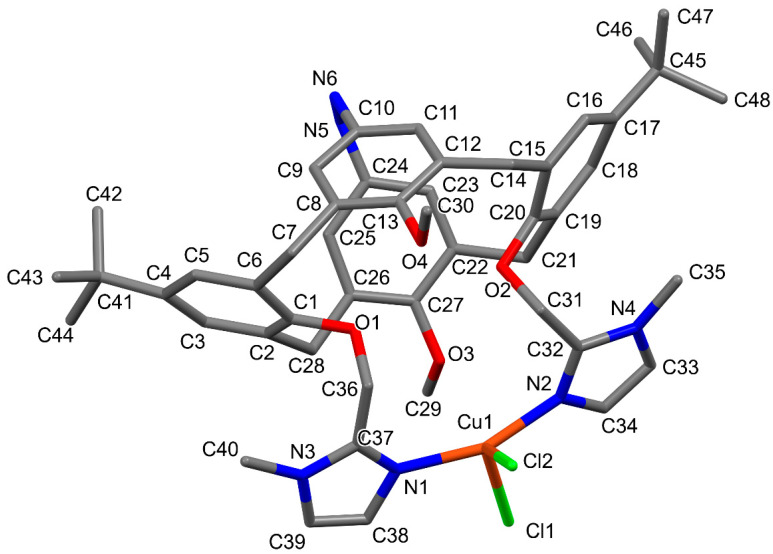
Molecular structure of **1a** with atom numbering. H atoms are omitted for clarity.

**Figure 2:**
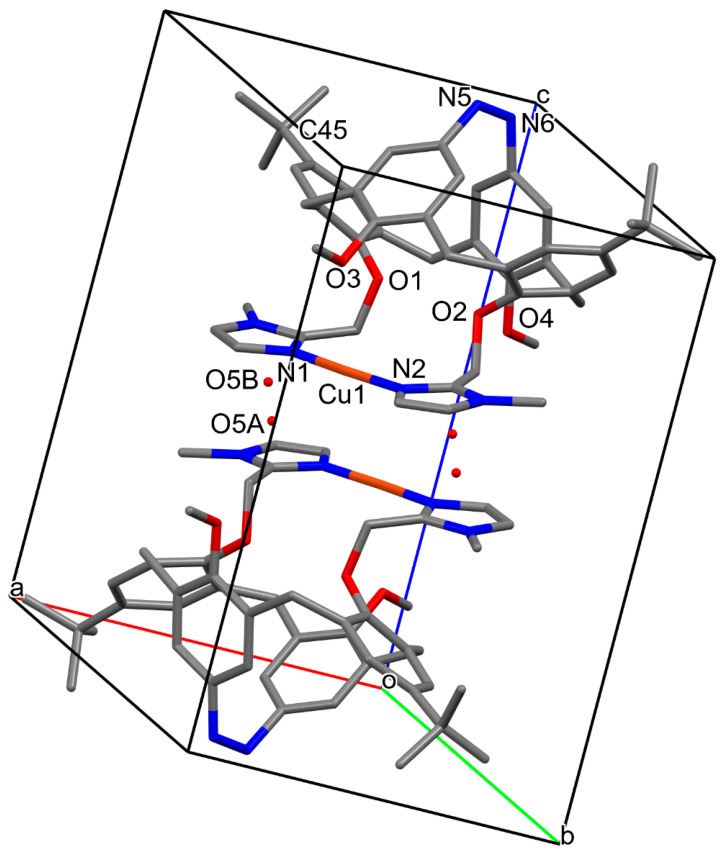
Unit cell content in **1b**. Only atoms discussed in the structure description are labelled. H-atoms are omitted for clarity. Only one orientation of the disordered *tert*-butyl group at C45 is shown.

Variation of both cations and anions (in the case of Cu^2+^ coordination) induces significant modifications in the mode of coordination. Copper coordination, which, when conducted in the presence of air, regardless of the anion used, occurs at the donor sites of both available methylimidazole units, is accompanied by a dehydrogenative coupling of two neighbouring aniline units, formation of an azo bridge at the large rim of the calixarene basket and, hence, closure of the calixarene basket from both sides. In both cases, using copper nitrate or chloride, the calix[4]arene cone conformation is strongly distorted upon coordination due to the formation of the azo bridge at the large rim (Scheme 2, Figure 1 and Figure 2). While two opposite phenyl units, bearing the methylimidazole coordination arms at the small rim, are leaned against the cavity axis (observation from the small towards the large rim), the remaining two phenyl units (bearing methoxy groups at the small rim) are gently leaned towards the central non-crystallographic *C*_2_ axis that runs through the centre of the calixarene basket. The nitrogen atoms of the azo bridge units are consequently endo-oriented, thus projecting the methoxy-groups outside of the cavity. Similar aerobic dehydrogenative coupling of aniline derivatives catalysed by Cu^2+^/pyridine to form azo compounds has been previously observed and reported [25]. A severely distorted tetrahedral coordination (CN = 4) sphere in which the divalent copper is coordinated by both available nitrogen atoms from methylimidazole coordination arms (N1 and N2) and with two chloride ions is observed in **1a** (Figure 1).

In **1b**, in the synthesis of which Cu(NO_3_)_2_ was used, copper adopts a linear coordination geometry with only two imidazole nitrogen atoms (N1 and N2) acting as donors. One methoxy oxygen from the calixarene core (O3) and one free hydroxide anion (occupying two different crystallographic positions with 75% (O5A) and 25% occupancy (O5B)), lay at distances from the copper cation that cannot be regarded as coordination contacts (Scheme 2 and Table 1). The hydroxide anion, clearly, balances the charge of the Cu^+^. Comparison of the Cu–N coordination bond lengths in **1a** and **1b** (Table 2) clearly reflects the difference in the oxidation state of the copper in these two complexes. This is further supported by the analysis of the CSD data for a chosen set of related structures. Among the 419 structures found in the current version of CSD [26] and containing Cu^+^ coordinated by just two nitrogen donors in a linear arrangement, the geometry of the coordination sphere astonishingly correlates with the one in **1b** (Table 2). At the same time, only a negligible number (24) of Cu^2+^ complexes (all of which are either polymeric or contain cumbersome co-crystallized chemical species) revealing linear NN 2-coordination were found in the database. There is one exception to this rule, where a 2-coordination of a Cu(II) was achieved on purpose for the first time [27]. When the database was searched for the tetracoordinated (NNOO) Cu^+^, very few structures revealed a Cu–O bond length of over 2.03 Å, let alone of ca. 2.7 Å, as found in **1b**. All these data leave no doubt that the copper in **1b** (unlike in **1a**) underwent a reduction from Cu^2+^ to Cu^+^ in addition to the dehydrogenative coupling of two neighbouring aniline units, which is observed in both complexes.

The molecular structure of **1b** is presented in Figure 2. Two complex molecules within the unit cell are mutually head-to-head oriented and related by a crystallographic inversion centre. Two imidazole units and Cu^+^ lay within the same plane, providing a flat roof of the calixarene molecule, unlike in the structure of **1a** (see Figure 1). In **1b**, these two planes, belonging each to one of the two neighbouring, mutually head-to-head, oriented molecules, are parallel, and lay at a distance of 3.259 (5) Å. Due to an offset of the two parallel planes (1.3 Å), copper ions lay at a mutual distance of 3.525 (5) Å, which can hardly imply any interaction between two neighbouring metal cations. However, the distance between the centroids of the two closest imidazole rings belonging to the neighbouring molecules [3.477 (6) Å] implies reasonably strong π–π interactions between them, which holds together the two roof-planes and thus guides the entire crystal packing (Figure 2).

Only one of the terminal *tert*-butyl groups (at C45) displays a positional disorder over two well-resolved crystallographic positions. In the final stages of the refinement, a solvent mask was applied to eliminate smeared electron density. No nitrate anion was observed in the structure.

In order to explore the dehydrogenation of the aniline units and formation of the azo bridge (**1a** and **1b**), as well as the reduction of the Cu^2+^ cation, involved only in the formation of **1b**, we have applied identical synthetic/crystallization procedures in the absence of air (Scheme 2). We observed no reaction of ligand **1** with CuCl_2_ over one week (copper salt remained non-solubilized and no colour change was observed). When the air was allowed to enter in contact with the reaction mixture, an instantaneous and intense darkening accompanied by the complete solubilization of the copper salt was observed. Slow evaporation of this solution at the RT did not produce single crystals even after the complete disappearance of the solvent. On the other hand, when Cu(NO_3_)_2_ was employed for the coordination of copper by compound **1** in the absence of air, we immediately observed an intense colour change (from light green to dark brown), resembling the reaction in the presence of air. No single crystals were produced from this reaction mixture after a pronounced slow evaporation in the absence of air, nor after the complete evaporation of the solvent.

Quantification of the reaction of ligand **1** into the azo products in the presence of Cu^2+^ was not possible in a direct manner since only small quantities of crystals were obtained. We attempted to get more information by analysing the mother liquors after the crystallization experiments. Their analysis by mass spectrometry disclosed a dominant presence of the intact di-aniline ligand **1**, indicating that the oxidation of ligand **1** is probably not quantitative and that the complexes formed with the azo bridge ligand selectively crystallized. All these findings suggest a synergistic effect of copper and oxygen in catalysing the formation of complexes **1a** and **1b**, as well as the decomposition of the nitrate and in-situ generation of oxygen, which then serves as a co-catalyser in the process. The fact that the Cu in our experiments ends up in a +1 or +2 oxidation state, depending on the anion, is due to the presence of the coordinating chloride anions in the case of **1a**, able to bind to copper and hence stabilize the +2 state.

**Figure 3:**
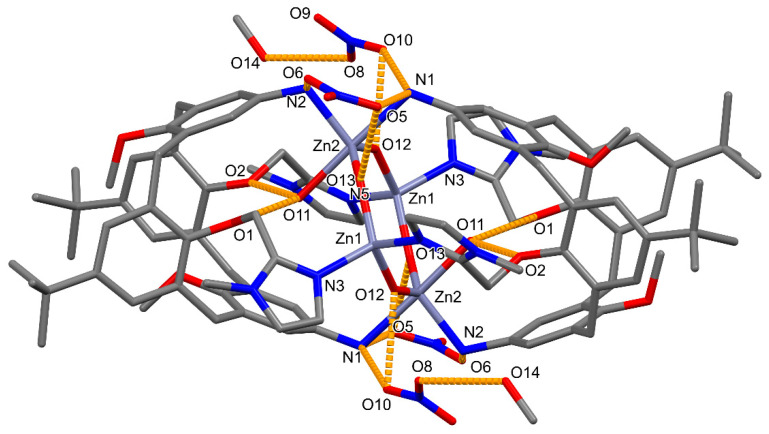
Molecular structure of **1c** with partial atom labelling and with H-bonds depicted. H-atoms are excluded for clarity. Only atoms involved in H-bonding and in Zn coordination are labelled, as well as the Zn ions. Only one (majoritarian) position of the disordered NO_3_^−^ group (N7, O5, O6, O7) is depicted for clarity.

**Figure 4:**
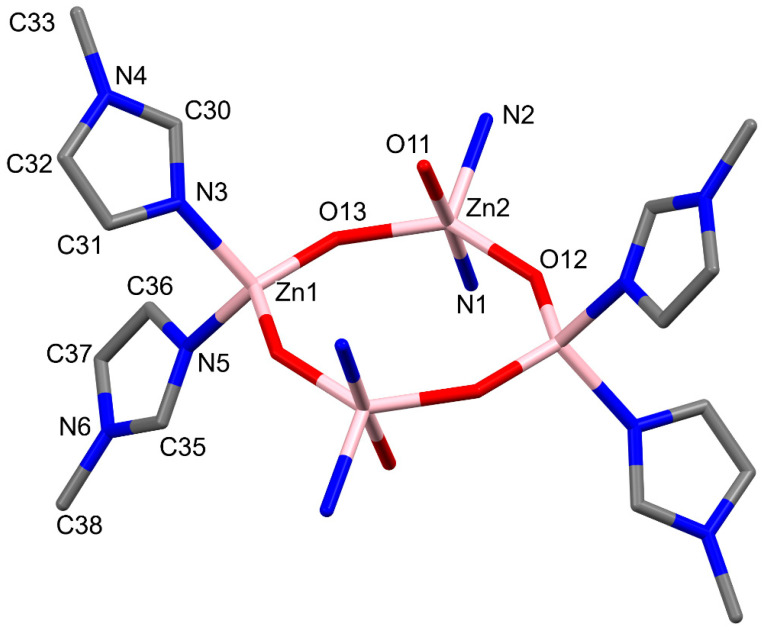
Coordination environment of four Zn ions with atom labelling. Only atoms from one asymmetric unit are labelled. H-atoms are omitted.

The crystallization was also tried with Zn^2+^ salts. An identical preparation procedure (as one of the copper complexes **1a** and **1b**) in which Zn nitrate was employed in lieu of the Cu^2+^ salts resulted in the formation of single crystals suitable for X-ray structure analysis. The Zn^2+^ complex **1c** crystallizes in a triclinic *P* − 1 space group with only one complex molecule per unit cell. Two methanol molecules are co-crystallized. The structure consists of a tetranuclear complex in which four zinc cations are interconnected via four hydroxyl groups to form a (-Zn-O)_4_ distorted octagon and provide, at the same time, four bridges holding together two, mutually tail-to-tail oriented, calixarene molecules in 1,3 alternate conformation via their imidazole nitrogen atoms (Zn1 and Zn1′, marked red in Scheme 2) and via their amino groups (Zn2 and Zn2′, marked blue in Scheme 2) (Figure 3 and Figure 4). Hence, Zn1 (as well as its symmetrical counterpart Zn1′) lies in the centre of a tetrahedral coordination sphere formed by two imidazole nitrogen atoms from two ligand molecules and two hydroxyl groups from the (-Zn-O)_4_ octagon, while Zn2 (as well as its symmetrical equivalent Zn2′) is five-coordinated, laying in a centre of a severely distorted trigonal bipyramid formed by two amino-nitrogens (emanating from two calixarene molecules), two hydroxyl groups (belonging to the (-Zn–O)_4_ octagon) and an additional water molecule (Scheme 2 and Table 3). The (-Zn-O)_4_ octagon is perpendicular to the (non-crystallographic) central axis running through the geometrical centre of two calixarene baskets. Zn1, Zn1′, O12, O12′, O13, and O13’ sit in a perfectly planar arrangement, while Zn2 and Zn2′ (five-coordinated, marked blue in the Scheme 2) are displaced from this *ls* plane for 0.288 (1) Å, laying on the opposite sides of the plane. The positive charge of the four Zn^2+^ is compensated by four hydroxyl groups from the (-Zn-O)_4_ octagon and by four NO_3_^−^ anions being firmly held by the main complex molecules by a series of H-bonds (vide infra). One of the NO_3_^−^ anions (at N7) is disordered over two well-resolved positions, with 0.75 and 0.25 occupancies, and with the common nitrogen atom. Both calix[4]arene units are in 1,3-alternate conformation, which optimizes the employment of the donor sites (amino-groups and imidazoles) in the Zn coordination. The conformation is further stabilized by several intramolecular H-bonds (Figure 3, Table 4). Amino group H atoms provide a tight and sensible connection between the complex molecule and all four counter-anions, which is further reinforced by the H-bonds connecting hydroxyl groups from the octagon (as donors) and oxygen atoms from the four nitrate anions. Two H-bonds, ensured by the donating potential of the coordinated water molecule (O11) towards the methoxy-oxygens O1 and O2 connecting the calixarene basket with the methylimidazole moieties, provide thermodynamic stabilization of the whole structure. Finally, there is an H-bond connecting the co-crystallized MeOH molecule with one of the nitrate anions.

### 2.2. NMR Studies

The X-ray structures reveal that ligand **1** can bind a metal cation [Cu^+^, Cu^2+^, Zn^2+^] with different modes of coordination in the solid state. So far, bis-imidazole calix[4]arene ligand analogues have shown a high selectivity for the coordination of Cu^+^ in a linear geometry [21,23]. In order to acquire a deeper insight into the coordination behaviour of calix[4]arene-based ligands in solution, we have investigated the coordination of ligands **1** and **2** with Zn^2+^ by NMR spectroscopy. Solubility issues during the titrations prevented the use of ligand **3**. It was, therefore, replaced by ligand **4** having two N-acetyl units on the large rim, but the titration also proved complicated for the same reason (vide infra).

Ligands **1** and **2** are not soluble in water at a mM concentration required for NMR studies, which were therefore conducted in CD_3_OD or CD_3_CN. First, ligand **1** was dissolved in CD_3_OD or CD_3_CN. In both cases, the ^1^H NMR spectrum is broad due to the methoxy-through-the-annulus rotation of the phenyl units. Sequential additions of one and two molar equivalents of Zn(NO_3_)_2_ or ZnCl_2_ were performed. In all four cases, the titration resulted in a significant modification of the initial ^1^H NMR spectrum, attesting to the interaction between the ligand and the Zn^2+^ cation (Figures S5–S8). HSQC NMR experiments allowed for the straightforward attribution of the well-defined peaks (Figures S9 and S10), particularly in the aromatic region of the spectrum. The addition of Zn^2+^ induces a significant shift for the two aromatic protons of the methylimidazole and a marked upfield shift for the protons on the aniline rings. These shifts are reminiscent of the ones observed earlier, obtained upon the linear coordination of bis-imidazole calix[4]arene ligands with the Cu^+^ cation [21,22,23]. They are due to the coordination of the two methylimidazolyl units to the cation and to the pinched conformation of the calixarene macrocycle orienting the aniline units towards the cavity. Unlike in this case of Cu^+^ coordination, and as previously observed for Na^+^ and K^+^, the coordination to the calixarene with the four oxygen atoms shapes the macrocycle in a straight cone conformation, yielding a very different ^1^H NMR pattern [23]. Finally, the new species was obtained after one equivalent of Zn^2+^ was added and the addition of an additional equivalent did not alter the spectra. Altogether, these data indicate a 1:1 coordination stoichiometry with the coordination of the two imidazole units. The coordination sphere might be completed by the counter anions, solvent molecules, or one oxygen atom of the methoxy groups. Finally, these data obtained in solution rule out that the mode of coordination of ligand **1** with Zn^2+^ is the one described in the solid state (complex **1c**), having four Zn ion vs. two ligand molecule stoichiometry and the calixarene in a 1,3-alternate conformation.

**Figure 5:**
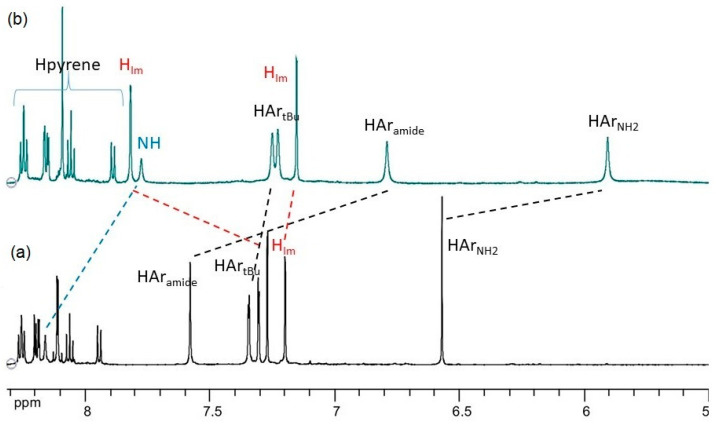
^1^H NMR spectra (600 MHz, 300 K, CD_3_CN) in the aromatic region of (**a**) **2** and (**b**) **2** with two equivalents of added ZnCl_2_.

The titration was repeated on the monopyrene conjugate **2** in CD_3_CN and monitored by NMR spectroscopy. The ^1^H NMR experiment reveals that the addition of ZnCl_2_ to a CD_3_CN solution of compound **2** is also responsible for a dramatic modification of some of the chemical shifts, especially for the aromatic protons. Upon coordination, most of the peaks accounting for the aliphatic protons become broad, anticipating a strong dynamic exchange (Figure 5 and Figure S11).

Indeed, as it was observed in the case of **1**, the calixarene conformation changes upon complexation with the Zn^2+^, as shown by the differences in the chemical shifts of the protons before and after complexation. Large changes in chemical shift upon complexation were observed for the protons of the imidazole units, attesting for the coordination of the Zn^2+^. Also, large negative induced shifts were observed for the aromatic protons of the aniline (HAr_NH2_) as well as for the aromatic protons of the phenylamide unit (HAr_amide_). This is also the consequence of the pinched conformation of the complex, in which the amino and amido groups of two phenyl units of the calix[4]arene not linked to the imidazole groups are oriented towards the cavity. NOESY NMR experiments clearly indicate a short contact between the two differentiated protons on the Ar_tBu_ units and the HAr_NH2_ or the HAr_amide_ protons, ruling out the existence of a 1,3-alternate conformation (Figure S12).

All these findings are in line with the formation of a Zn^2+^ complex, in which the cation is bound to two nitrogen atoms of the methylimidazolyl units, leaving the calixarene in a strongly pinched conformation. In solution, the sphere of coordination around the Zn^2+^ is likely completed by the chloride anions and/or solvent molecules.

Finally, ligand **4**, a model for ligand **3** devoid of the pyrene units, was also affected by the addition of Zn^2+^. The first 0.5 equivalent did modify the ^1^H NMR spectrum, as for the other ligands, but an addition of more Zn^2+^ caused the precipitation of the ligand or other species (Figure S13).

The assignment of the ^1^H spectra of the complex formed with ligand **2** was based on COSY, HSQC, and HMBC spectra (Figures S14–S21).

In conclusion, the NMR characterization in solution (CD_3_CN and CD_3_OD) proved that the bis-imidazole calixarene ligands can coordinate Zn^2+^ with two methylimidazolyl units, placing the calixarene in a pinched conformation similar to the one observed in the solid state, in copper complexes **1a** and **1b,** or to the previously reported bis-imidazole calix[4]arenes upon linear 2-coordination with the Cu^+^ cation [21,22,23].

### 2.3. Fluorimetric Titrations

**Figure 6:**
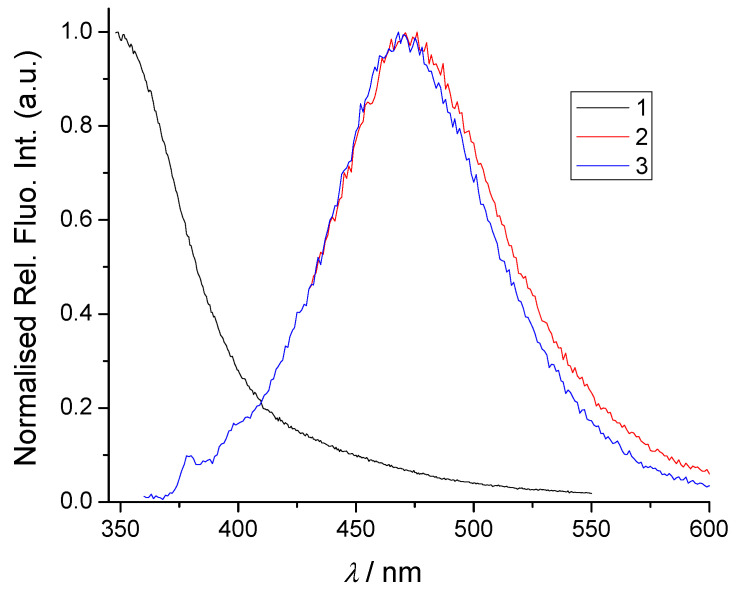
Normalised fluorescence spectra of studied **1** (*λ*_exc_ = 300 nm, water), **2** (*λ*_exc_ = 350 nm, water), and **3** (*λ*_exc_ = 350 nm). Normalisation was performed by dividing the complete spectrum by the maximal intensity of emission.

We studied the interaction of **1**, **2,** and **3** with a series of transition metal cations in water to test the coordination affinity and spectroscopic response in biorelevant conditions of these potentially powerful DNA/RNA and mononucleotide binders [5,24]. The set of cations for this study was carefully chosen because of this objective [20]. Cu^+^, Cu^2+^, Zn^2+^, and Co^2+^ are chosen as the representatives of the biologically important metals [28], whereas Cd^2+^ is a representative of highly toxic pollutants [29]. All the studied compounds are intrinsically fluorescent (Figure 6), thus we presumed that metal coordination could cause rigidification of a calix[4]arene cavity [21], which may influence the electronic properties of calix[4]arene fluorophore of 1 and also the intramolecular pyrene interaction with the calix[4]arene core, resulting in pyrene fluorometric response (in the case of **2** and **3**, vide infra) [24]. Thus, fluorometric titrations would allow determination of binding constants for **1**–**3**/cation complexes formed.

**Figure 7:**
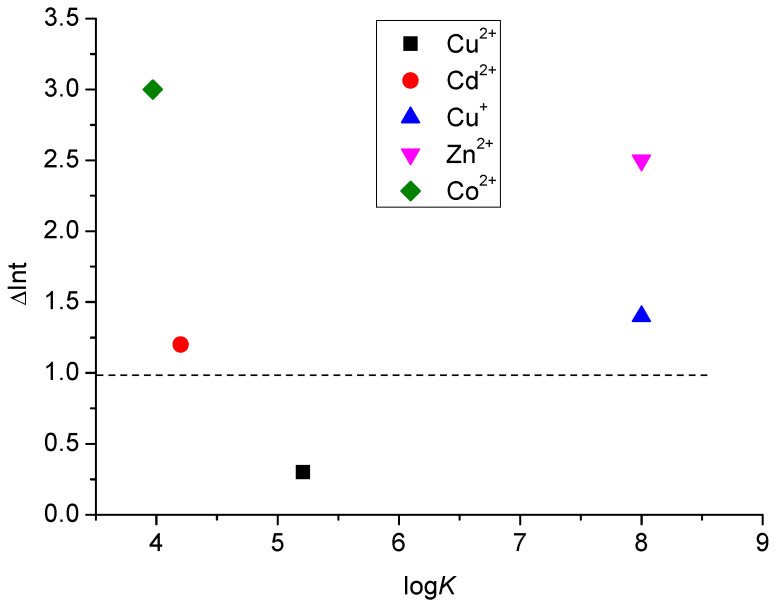
Comparison of stability constants and relative intensity values (Δ*Int* = *Int*_(100% complex)_/*Int*_0_) from fluorometric titrations of **1** with different cations in water.

Indeed, this study has shown that fluorescence emission spectra of **2** and **3** were systematically quenched upon any cation addition (Figures S27–S34). For comparison, the same titrations were performed with **1**, taking advantage of its intrinsic fluorescence (emanating from the calixarene core, as demonstrated in our previous study) [5]. The fluorescence of **1** was sensitive to the type of the metal cation added to the solution (Figure 7), whereby the addition of Cu^2+^ caused emission quenching (Figure S22), while the addition of all other tested cations from the series caused an emission increase (Figures S23–S26).

The metal cation induced quenching of pyrene emission in **2** and **3** could, theoretically, be of a dynamic or static nature [30]. However, fluorescence lifetime experiments revealed that the fluorescence lifetime of both **2** and **3** did not change upon the addition of Cu^2+^ (Tables S2 and S3, Figures S36 and S37), supporting a dominant role of the static quenching mechanism [18,31]. In all titrations, the concentration of dye is very low (5 µM), and the cation concentrations never exceeded 50 µM; therefore, the dynamic quenching (resulting from diffusive encounters between the fluorophore and quencher during the very short lifetime of the excited state) [18,31] is very unlikely to be an overriding occurrence. Therefore, for the fluorometric titrations in water (Table 5), the static quenching is a dominant binding event.

The processing of the titration data (complete spectra) employing a multivariate non-linear regression method (Specfit program (v0.5.0b2)) [32,33] resulted in the best fit for the 1:1 stoichiometry complex (${ML}^{n+}$) for all tested cations, yielding respective binding constants and emission change percentages (Table 5). Analysis of the data in Table 5 revealed that the affinity of **1** towards any cation from the series is lower than that obtained for pyrene-containing derivatives **2** and **3**. This indicates that the complexation of **2** and **3** with cations is hydrophobically driven by the presence of pyrene moieties; aromatic stacking interactions between pyrene(s) and phenyl units that form the calix[4]arene core favour the binding affinity. The only exception is the high affinity of **1** towards Cu^+^, with the stability constant estimated at log*K* > 8 (Figure S24), which was expected and confirms our previous findings as well as the results of SCXRD (vide supra) [22].

The emission response of **1** is not directly correlated with stability constants. For instance, the most stable complex **1**/Cu^+^ is much less emissive than the analogous complexes of other cations from the series (Figure 6, Table 5). This is not surprising, since the emissive properties of a fluorophore are directly dependent on cation size, electron density, directionality of coordination, and complex interplay with excited-state relaxation mechanisms, whereas the binding constant is not proportionally related to the mentioned properties.

The presumption is that the insertion of a metal cation into the calix[4]arene core and engagements of the methylimidazole coordination arms in the cation entrapment causes rigidification of the macrocyclic basket and displacement of pyrene units away from it, which necessarily diminishes the contact of pyrene unit(s) with their exciplex partner {calix[4]arene core} and results in the signal quenching. In such a scenario, there are several factors determining the efficiency of the complex formation, which are all to be considered. Firstly, larger cations are less solvated than smaller ones (like, for instance, Cu^2+^) and are therefore more easily approached by ligands due to the lower cation desolvation penalty [34]. Also, the formation of the complex in water depends on matching the cavity size of the ionophore and the effective diameter of the first hydration shell of metal ion [31]. Hence, in this case, smaller cations fit better into the calixarene binding site. Finally, the Lewis acidity of the cations influences their affinity towards the only available N-donor sites.

Detailed analysis of Table 5 reveals two different scenarios. The first is titration ending at calix/cation ratio = 1:1 (Figures S24, S30, S32, and S34), whereby most of the emission change happens below the 1:1 ratio, suggesting the eventual formation of other stoichiometries; for instance, at an excess of calix over metal cation, calix/cation = 2:1 complex could be formed. Fitting of titration data by the multivariate fitting procedure of the Specfit program to calix/cation ratio = 2:1 gave similar quality of a fit as 1:1 stoichiometry; however, at such low concentrations we could not prove the presence of both stoichiometries. Since all other data and literature references suggest the dominance of 1:1 calix/cation complex, we attributed to such systems a very high overall binding constant (log*K* > 8). This is observed for pyrene-devoid parent ligand **1** in interaction with Cu^+^ and Zn^2+^, as well as for pyrene-containing analogues **2** and **3** with Zn^2+^ and Co^2+^. The emission of fluorophores is either increased (**1**) or only partially quenched (**2**, **3**), the latter additionally supporting the static quenching mechanism. The second scenario involves titrations of **1**, **2,** and **3** with Cu^2+^ and Cd^2+^, which end at the excess of a cation over dye (ratio >> 2), allowing accurate processing of titration data by the multivariate analysis least square program in Specfit, which, for all cases, gave the best fit for the formation of the 1:1 dye:cation complex. In such cases, the stability constants of the complexes of the mono-pyrene derivative **2** and its bis-pyrene analogue **3** with Cu^+^, Cu^2+^, and Cd^2+^ are within the same order of magnitude.

As the counter-anion may, generally speaking, play an important role in metal coordination [35], we have performed a series of titrations alternating chloride and perchlorate salts (Figures S31–S34). The obtained results, however, revealed only a negligible impact of the anion on the binding affinity in the case of our compounds (Table 5). Also, the addition of the tetra-methylammonium chloride to **2** (Figure S35) had no apparent effect on the fluorescence emission, stressing the decisive role of a metal cation in all fluorometric titrations.

Referent compound **1** showed a similar binding affinity for Cu^2+^ as **2** or **3**. However, in comparison to pyrene-containing analogues **2** and **3**, the pyrene-devoid ligand **1** showed a much lower affinity for Cd^2+^; whereas, intriguingly, for the Cu^+^ cation, the selectivity was opposite [log*K*(**1**) >> log*K*(**2,3**)]. It appears that metal cation binding to **1** in comparison to **2** and **3** is comparable only for some cations; only for Cd^2+^ are the affinities of **2** and **3** clearly larger than for **1**. Such differences in cation binding between **2** and **3** versus **1** are likely a consequence of different binding sites of a cation and/or involvement of pyrene subunits in complex formation. Important to note is that these derivatives can be used as a reversible fluorescence sensor towards some cations according to moderate binding affinities and the possibility of detecting the forward/reverse reactions by turning fluorescence on/off, depending on cation concentration by simple fluorescence signal monitoring [36,37]. For instance, calixarene **1** can be applied as a reverse chemosensor for Co^2+^ and Cd^2+^ (log*K* = 3–4, fluorescence on upon complexation) and calixarenes **2** and **3** for Cu^2+^, Cu^+^, and Cd^2+^ (log*K* = 5–6, fluorescence off in a complex).

### 2.4. Molecular Modelling

**Figure 8:**
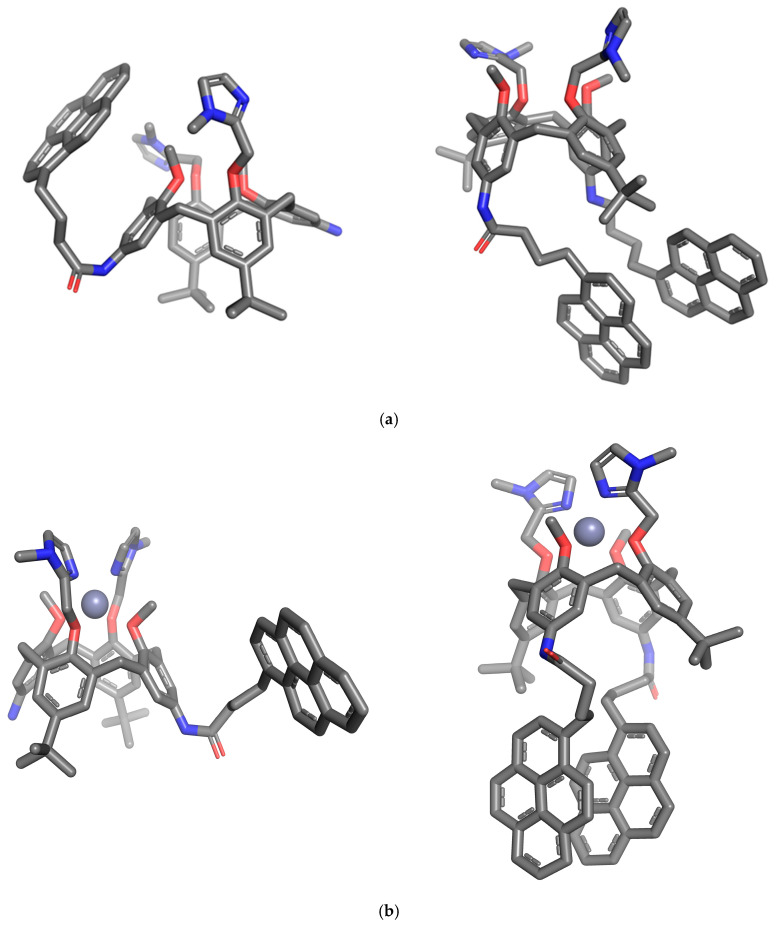
MD structures of (**a**) calix[4]arene-pyrene conjugates **2** (**left**) and **3** (**right**) and (**b**) their Zn^2+^-complexes, **2**/Zn^2+^ (**left**) and **3**/Zn^2+^ (**right**); after 100 ns at *T* = 303 K. Water molecules and chlorine counterions are omitted for clarity.

We have performed molecular dynamic simulations of **2** and **3** and their Zn^2+^-complexes in water to obtain an insight into the most probable conformational modifications of the pyrene calixarene conjugates upon metal coordination. The purpose was to gain an idea of the structural and physico-chemical basis of the observed emission quenching of these compounds in presence of the metal cations. Computational details are given in Supplementary Material in Computational simulations protocols section and different conformations were analysed during the minimization, heating, equilibration and production MD step (Supplementary Materials Figure S38) [38,39,40,41,42,43,44,45,46,47,48,49,50,51]. The structures obtained after 100 ns MD simulation (Figure 8a) show that in conjugate **2** the pyrene unit experiences aromatic stacking interactions with the phenyl moiety from the calix core, which strongly supports the hypothesis of exciplex formation. Its bis-pyrene analogue **3**, after 100 ns of the MD simulation, was characterized by two parallel, face-to-face oriented and closely spaced pyrene arms, with distances between carbon atoms of the neighbouring units in the range of 3–4 Å. This conformation strongly supports the hypothesis of pyrene excimer formation [18,31]. Calix[4]arene conformation of mono-pyrene derivative **2** is being preorganized for the metal ion entrapment at the small rim by its two methylimidazole coordination arms. The *tert*-butyl bearing phenyl units are close-to-parallel to each other, while the remaining two amino/amido groups bearing phenyl units are strongly leaned, projecting their amino/amido groups outwards of the cavity. This is a typical pinched conformation of the calix[4]arene basket, which was also observed in the solid state for the metal complexes of the pyrene-devoid parent compound **1**, as well as in solution, for both **1** and **2**, and their Zn^2+^ complexes. However, in all these examples (both in solution and in solid state), the leaned and parallel phenyl units were inverted, as compared to the results obtained with molecular simulations for the monopyrene conjugate. In the bis-pyrene derivative **3,** a cone conformation of the calixarene basket is obtained, and imidazole coordinating arms are projected outside the cavity.

In both free ligands, the groups grafted at both large and small rims rotate freely in solution. Upon the addition of the Zn^2+^, however, methylimidazole coordination arms grafted at the small rim orientate inwards and encapsulate the cation in the binding site, between the two methylimidazole nitrogen donors. The NMR and X-ray studies have revealed that the preferred binding site for both Zn^2+^ and Cu^2+^/Cu^+^ is the one provided by the nitrogen donor atoms of the methylimidazole units. DFT optimizations and MD simulations of **2**/Zn^2+^ and **3**/Zn^2+^ in water reveal a complex with Zn^2+^ firmly encapsulated between the methylimidazole nitrogen atoms and four methoxy oxygen atoms. However, the distances between Zn^2+^ and N-/O-donor atoms show that Zn^2+^ is coordinating to methylimidazole nitrogen atoms (*d* = 2.1 Å), while the distances towards methoxy oxygen atoms are too large (*d* = 2.5 Å) to be considered as coordination bonds.

The simulations also clearly reveal that the coordination of the Zn^2+^ by the amino and/or amide groups at the large rim is not possible, since their mutual distances exceed 9 Å and hence are too large to enable encapsulation of a cation without significant disruption of very strong and dominant π–π aromatic stacking interactions.

Importantly, cation binding can be accompanied by significant structural rearrangements of the ligand structure and loss of aromatic stacking interactions. For instance, the pyrene leg of the monopyrene conjugate **2** undergoes displacement away from the methylimidazole binding site upon the addition of the Zn^2+^ to the system. This has an impact on the pyrene–calixarene aromatic stacking interactions in **2** and decreases the exciplex signal, as demonstrated with the fluorometric titrations. On the other hand, in the bis-pyrene conjugate **3**, pyrene–pyrene stacking interactions are only marginally influenced by the cation binding on the opposite rim (Figure 8b), which suggests that Zn^2+^ complexation only partially disrupts the pyrene-excimer, yet still reduces the excimer emission.

## 3. Conclusions

A series of bis-imidazole-calix[4]arene derivatives **1**–**4** showed a remarkable ability to accommodate both monovalent and divalent transition metal cations, as proven by NMR, single crystal X-ray diffraction experiments, as well as by fluorometric measurements further supported by computational simulations. As we have reported previously [5,24], our specially designed calixarene-based ligands strongly interact with ds-DNA/RNA, but only those among them bearing a permanent positive charge. The coordination of neutral ligand to metal cations with high-affinity constants is an alternative to introduce positive charges and potentially enhance the association of these binders to polynucleotides. The NMR experiments, as well as the single crystal X-ray structures and MD simulations, have confirmed that the bis-methylimidazole coordination site is the primary location for cation binding. A very particular X-ray structure of the Zn complex of the ligand **1** is the only example in which methylimidazole nitrogen atoms together with the amino nitrogens from the opposite calixarene rim are involved in the formation of a coordination site. It is also a unique example in this study in which calix[4]arene exists in a 1,3-alternate conformation. In solution, this ligand, whether alone or coordinated by Zn^2+^, as is the case with its derivatives, adopts a well-known pinched cone conformation.

Fluorometric measurements and NMR studies have revealed that anions have little or no impact on the metal cation binding affinity of our systems (both pyrene-armed and pyrene-devoid). X-ray structures of copper complexes of the pyrene-devoid parent ligand **1** have revealed an anion impact on the chemistry of coordination and composition of the first coordination sphere, but confirmed as well that the anion does not affect the binding mode itself. Unexpectedly, we have observed that during the crystallization of ligand **1** in the presence of Cu^2+^ and oxygen, a dehydrogenation of two neighbouring aniline units at the small rim of the calix[4]arene **1** occurred, leading to the formation of an azo bridge. A similar Cu^2+^ catalysed dehydrogenation of aniline substrates with aromatic N-donor ligands was previously reported [31]. It emphasises the potential of our bis-imidazole calixarene structures to be explored as potential ligands in catalysis for a variety of redox reactions.

In the biorelevant aqueous solution, the comparison of fluorometric titrations of referent calix[4]arene with its analogues having one or two pyrene units grafted at the opposite (large) rim revealed a very different (10 µM to 10 nM) affinity towards transition metal cations and, more importantly, the strong impact of pyrene on the binding affinity towards some cations. The presence of pyrene arm(s) in **2** and **3** significantly diminished the affinity revealed by referent **1** towards Cu^+^, but strongly increased the affinity towards divalent Co^2+^ and Cd^2+^ cations. Since the counter-anion plays only a marginal role, such change in selectivity is attributed to the intramolecular interaction of pyrene(s) with the calix[4]arene-imidazole system, sterically controlling the metal cation binding site. Moreover, the fluorometric response of some studied derivatives, particularly of **1,** was strongly sensitive to the cation type. Derivatives **1**–**3** can also be applied as reverse chemosensors for some cations in biologically relevant conditions, which is particularly important for imaging metals in biology. Obtained results in detail characterised the biorelevant metal cation binding abilities of bioactive pyrene-calix[4]arene-imidazole systems, thus equipping them with an additional positive charge and the bioactivity of particular metal cations. Although studied compounds have only moderate solubility in water, they are well-soluble in more lipophilic solvents and bind strongly to biomacromolecules (ds-DNA, see Section 1). Thus, in eventual future biological applications, they would, due to hydrophobicity, very likely bind and accumulate in proteins/membranes or bind to ds-DNA and deliver the chosen metal cation. For instance, Cu^2+^ delivered by calixarene into ds-DNA could cleave DNA backbone. Mentioned possibilities support further studies of these metal complexes’ interactions with biorelevant targets.

## 4. Materials and Methods

### 4.1. Chemicals for Synthesis and Physicochemical Investigations

Solvents used in syntheses, crystallizations and binding studies were Milli Q water, dimethyl sulfoxide (DMSO)(Alfa Aesar, Karlsruhe, Germany), methanol (99.8%, Kemika, Zagreb, Croatia), acetonitrile (99.5%, Kemika, Zagreb, Croatia), deuterated acetonitrile, and deuterated methanol (Euroisotop, 99.99%) and were used without further purification. Freshly dried DCM was used for the synthesis of complex **4**. The salts used for the investigation of calixarene complexation were Cu(MeCN)_4_PF_6_ (97%, Sigma Aldrich, Darmstadt, Germany), CuCl_2_ (99.99%, Sigma-Aldrich, Darmstadt, Germany), CuI (99.9%, Sigma-Aldrich, Darmstadt, Germany), CdCl_2_ × 2.5 H_2_O (99.8%, Sigma-Aldrich, Darmstadt, Germany), CoCl_2_ × 6 H_2_O (99.99%, Sigma-Aldrich, Darmstadt, Germany), ZnCl_2_ (98%, Merck, Darmstadt, Germany), Cu(ClO_4_)_2_ × 6H_2_O (98%, Sigma-Aldrich, Darmstadt, Germany), Cd(ClO_4_)_2_ × H_2_O (99.999%, Merck, Darmstadt, Germany), Co(ClO_4_)_2_ × 6H_2_O (99.999%, Merck, Darmstadt, Germany), Zn(ClO_4_)_2_ × 6H_2_O (99%, Sigma-Aldrich, Darmstadt, Germany), and Zn(NO_3_)_2_ × 4H_2_O (99%, Sigma-Aldrich, Darmstadt, Germany). Tetramethylammonium chloride (TMACl) was purchased from Sigma-Aldrich (Darmstadt, Germany) (≥98%). 

### 4.2. Synthesis

Syntheses of ligands **1**, **2,** and **3** are described in our previous reports [22,24]. The synthesis of compound **4** was inspired by the recently reported synthesis of N-ethyl-N-phenylacetamide (step I) [52] (Figures S1–S4). Calix[4]arene **1** (66 mg, 0.08 mmol) was dissolved in dry DCM (8 mL), Et_3_N was added (4 eq, 32 mmol, 46 μL), and the mixture was put under Ar and left stirred at 0 °C for 15 min. Acetyl chloride (3 eq, 0.24 mmol, 18 μL) was added, and the reaction mixture was brought back to the RT and left stirred overnight. Water was added (10 mL), pH checked (pH = ca 8), aquatic phase extracted with DCM (3 × 5 mL), organic phases combined and washed with water (2 × 10 mL), dried over Na_2_SO_4_, filtered and evaporated under reduced pressure to yield 62 mg (88%) of pale-yellow powder of **4**. ^1^H NMR [600 MHz, CD_3_CN + Cu(MeCN)_4_PF_6,_ 1eq], δ (ppm): 7.69 (s, 2H, NH), 7.38 (br s, 2H, H_Im_), 7.19 (s, 4H, H_Ar_), 7.10 (br s, 2H, H_Im_), 6.37 (bs, 4H, H_Ar_-NH-), 5.31 (br s, 4H, CH_2_-O-), 3.72 (br s, 4H, Ar-CH_2_), 3.62 (br s, 6H, O-CH_3_), 3.05 (d, 4H, Ar-CH_2_), 2.84 (br s, 6H, N-CH_3_), 1.84 (s, 6H, N-CO-CH_3_), 1.34 (s, 18H, tBu). ^13^C NMR [125 MHz, CD_3_CN + Cu(MeCN)_4_PF_6,_ 1eq], δ (ppm): 168.8, 154.3, 148.1, 136.7, 134.3, 126.9, 121.0, 66.9, 62.9, 34.9, 31.8, 31.3, 30.3, 24.2. HRMS (ESI+, MeOH) *m*/*z*: (**4**: C_52_H_62_N_6_NaO_6_; calcd. for [**4** + Na]^+^: 889.4623, found for [**4** + Na]^+^: 889.4599).

### 4.3. Fluorimetric Studies

All fluorescence measurements were performed on a Cary Varian Eclipse fluorimeter and an Edinburgh FS5 fluorimeter (Edinburgh Inst., Edinburgh, UK) at 25 °C in water, taking care that the absorbance of a sample at the excitation wavelength was <0.05 to avoid any impact of the inner filter effect. All spectrophotometric data were processed using Origin 7.5 or Specfit to obtain graphical curves and calculation of binding constants.

Fluorescence lifetimes for conjugates **2** and **3** and their Cu^2+^ complexes were measured using a 340 nm emitting picosecond pulsed diode laser as the light source in the time-correlated single-photon counting (TC-SPC) mode. Emission decay was detected with a 15 nm emission-slit bandwidth. The duration of the pulse was ≈800 ps. A 5000 kHz (every 200 ns) pulse was generated. Fluorescence signals at 475 nm were monitored over 1023 channels with a time increment of 500 ns/1024 channels. The decays were collected until they reached 3000 counts in the peak channel. Instrument response functions (IRFs) were measured from the scatter of an aqueous suspension of Ludox (suspension of silica gel in H_2_O) at the same excitation wavelength. Absorbances of the samples at 340 nm were <0.1. Before each measurement, the solutions were purged with a stream of argon for 20 min. A reconvolution-fit, as a sum of exponentials, was performed using Fluoracle software (Version 2.11.2), implemented with the instrument. The quality of the decay fits was assessed by the calculated values of the reduced χ^2^ and Durbin–Watson parameters and visual inspection of the weighted and autocorrelated residuals.

All measurements were performed in standard quartz cuvettes (Hellma Suprasil QX, 1 cm × 1 cm cross-section) (Müllheim, Germany) fitted with screw caps in Milli Q water at room temperature (25 °C).

### 4.4. Crystallizations

**1a**. A solution of **1** in MeOH (1 mL, 0.016 mmol/mL, 0.016 mmol) was added dropwise to the equimolar stirring solution of CuCl_2_ (2 mL, 0.008 mmol/mL, 0.016 mmol, MeOH), and the reaction mixture was left stirred over 20 min. The reaction mixture became dark green soon after the first drops of the ligand solution were added. The solution was left to evaporate slowly at RT, to yield, after five days, clear yellow prisms of **1a** when the mother liquor was almost completely evaporated.

**1b**. A solution of **1** in MeOH (1 mL, 0.016 mmol/mL, 0.016 mmol) was added dropwise to the equimolar stirring solution of Cu(NO_3_)_2_ (2 mL, 0.008 mmol/mL, 0.016 mmol, MeOH), and the reaction mixture was left stirred over 20 min. The reaction mixture became dark green soon after the first drops of the ligand solution were added. The solution was left to evaporate slowly at RT, and after six days, clear colourless prisms of **1b** appeared when the mother liquor was almost completely evaporated.

**1c**. A solution of **1** in MeOH (2 mL, 0.024 mmol/mL, 0.048 mmol) was added dropwise to the equimolar stirring solution of Zn(NO_3_)_2_ (1 mL, 0.048 mmol/mL, 0.048 mmol, MeOH), and the reaction mixture was left stirred over 20 min. No colour change was observed. The reaction mixture was left to slowly evaporate at RT, which yielded, after three days, colourless prisms of **1c**.

### 4.5. X-ray Diffraction Studies

Crystal data, data collection and refinement parameters are summarized in Table S1. Data collections for all samples were performed at LT (100 K) with the Copper radiation (*λ* = 1.54183 Å) at the XtaLAB Synergy Dualflex diffractometer. Data reductions and cell refinements were carried out using CRYSALIS PRO software (v1.171.38.46) [53], and standard multi-scan absorption correction was applied. Structures were solved by direct methods with SHELXT [54] and refined by a full matrix least-squares refinement based on *F*^2^ with SHELXL [55]. Molecular illustrations were prepared with MERCURY [56]. Mentioned software for the structure solution, refinement, and molecular visualization is included in the WinGX interface [57].

In **1a** and **1b**, all H atoms were included in their geometrically calculated positions and refined according to the riding model. In **1c**, H atoms possibly involved in H-bonding (amino groups hydrogens, coordinated water and hydroxide hydrogens, and co-crystallized methanol molecule hydrogen H14A) were located from the difference map and refined freely.

In the final stages of all three refinements, a BYPASS procedure (solvent mask) [58] included in the OLEX package (v1.5) [59,60] was applied in order to exclude from further refinement calculation the area of non-attributable and irrelevant electron density. The structure of **1a** is somewhat ill-refined due to a low quality of the crystal.

CCDC 2364990, 2364991, and 2364992 contain the supplementary crystallographic data. These data can be obtained free of charge via http://www.ccdc.cam.ac.uk (accessed on 14 October 2024), or from the Cambridge Crystallographic Data Centre, 12 Union Road, Cambridge CB2 1EZ, UK; fax: (+44)-1223-336-033; or e-mail: deposit@ccdc.cam.ac.uk.

### 4.6. NMR Studies

NMR spectra were recorded in CD_3_CN or CD_3_OD on Bruker AV 600 MHz or Bruker Avance ll 500 spectrometers. Chemical shifts (δ) are expressed in ppm. Details are given in the Supplementary Materials.

## Figures and Tables

**Table 1:** Geometry of the coordination spheres around copper in **1a** and **1b**.

**Bond (Å)**	**1a**	**1b**
Cu–N1	1.967 (11)	1.884 (3)
Cu–N2	1.961 (13)	1.884 (3)
Cu–Cl1/03	2.362 (4)	2.774 (2)
Cu–Cl2/O5A (O5B)	2.286 (4)	2.766 (7) [2.608 (8)]
**Angle (°)**	**1a**	**1b**
N1–Cu–N2	159.4 (4)	173.67 (11)
N1–Cu–Cl1	97.6 (4)	
N1–Cu–Cl2	95.0 (3)	
N2–Cu–Cl1	93.1 (4)	
N2–Cu–Cl2	94.3 (3)	
Cl1–Cu–Cl2	121.34 (17)	

**Table 2:** Geometry of the Cu^+^ and Cu^2+^ NN- and NNOO-coordination spheres in CSD.

Struct. Fragment	N_sf_	Cu–N (Å)	Cu–O (Å)	N–Cu–N (°)
N–Cu^+^–N	419	1.88 (4)		173 (7)
N–Cu^2+^–N	24	1.93 (7)		
NNOO–Cu^+^	849	1.97 (4)	1.94 (9)	

**Table 3:** Geometry of the coordination spheres around Zn1 and Zn2 in **1c**.

**Bond (Å)**	**Zn1**	**Zn2**
Zn–N3/N1	2.039 (2)	2.340 (2)
Zn–N5*i*/N2*i*	2.047 (2)	2.114 (2)
Zn–O12	1.919 (1)	1.923 (1)
Zn–O13/O13*i*	1.919(2)	1.925(2)
Zn–O11	-	2.184 (2)
**Angle (°)**	**Zn1**	**Zn2**
N3/N1–Zn–N5*i*/N2 *i*	103.62 (9)	82.92 (8)
N3/N1–Zn–O12	104.24 (9)	92.34 (8)
N3/N1–Zn–O13/O13 *i*	110.60 (9)	90.92 (9)
N1–Zn–O11		177.88 (8)
N5*i*/N2*i*–Zn–O12	110.64 (8)	112.48 (9)
N5*i*/N *i*–Zn–O13/O13*i*	103.43 (9)	111.54 (9)
N *i*–Zn–O11		98.78 (8)
O12–Zn–O13/O13*i*	122.86 (8)	135.92 (8)
O12–Zn–O11		88.18 (8)
O13*i*–Zn–O11		87.28 (9)

Symmetry operation “*i*” stands for 1 − *x*, 1 − *y*, 1 − *z*.

**Table 4:** Hydrogen bonds in **1c**.

*D*-H⋯*A*	*D*-H (Å)	H⋯*A* (Å)	*D*-H⋯*A* (Å)	*D*-H⋯*A* (°)	Sym. on *A*
N1–H1c ⋯ O5*i*	0.84 (4)	2.42 (4)	3.128 (4)	142 (3)	1 − *x*, 1 − *y*, 1 − *z*
N1–H1d ⋯ O10*i*	0.83 (4)	2.34 (4)	3.054 (5)	145 (3)	1 − *x*, 1 − *y*, 1 − *z*
N2–H2b ⋯ O6	0.87 (4)	2.07 (4)	2.922 (4)	169 (4)	
O11–H11a ⋯ O1*i*	0.89 (5)	1.97 (5)	2.850 (3)	166 (4)	1 − *x*, 1 − *y*, 1 − *z*
O11–H11b ⋯ O2*i*	0.75 (4)	2.13 (4)	2.867 (3)	169 (3)	1 − *x*, 1 − *y*, 1 − *z*
O12–H12a ⋯ O10*i*	0.78 (5)	2.13 (4)	2.858 (4)	155 (4)	1 − *x*, 1 − *y*, 1 − *z*
O13–H13 ⋯ O5	0.69 (4)	2.27 (5)	2.957 (4)	173 (4)	
O14–H14a ⋯ O8	0.84 (6)	2.09 (6)	2.840 (4)	149 (5)	

**Table 5:** Fluorometrically determined binding constants (log*K*) ^a^ and emission change (Δ*int* ^b^, in parentheses) for **1**, **2**, and **3**. Performed in water.

Cation	Anion	1	2	3
Cu^2+^	Cl^−^ ClO_4_^−^	5.21 ± 0.06 (0.3) nd	5.41 ± 0.03 (0.3) 6.07 ± 0.07	5.29 ± 0.02 (0.4) nd
Cu^+^	I^−^	^c^ >8 (1.4)	5.93 ± 0.04 (0.3)	5.69 ± 0.02 (0.5)
Cd^2+^	Cl^−^ ClO_4_^−^	nd 4.2 (1.2)	6.63 ± 0.06 (0.4) 5.63 ± 0.1	6.36 ± 0.06 (0.6) nd
Co^2+^	Cl^−^ ClO_4_^−^	3.97 ± 0.07 (3) nd	^c^ >8 (0.8) ^c^ >8	^c^ >8 (0.6) nd
Zn^2+^	Cl^−^ ClO_4_^−^	^c^ >8 (2.5) nd	nd ^c^ >8 (0.1)	nd ^c^ >8 (0.2)

^a^ Titration data were analysed by multivariate analysis program Specfit; ^b^ Δ*Int* = *Int* _(100% complex)_/*Int*_0_; ^c^ end of titration at *r*_[dye]/[cation]_ = 1 did not allow accurate processing of titration data for calculation of binding constant, and thus overall binding affinity was estimated at log*K* > 8; nd stands for “not determined”.

## Data Availability

All data are contained within this manuscript and the Supplementary Materials file.

## Supplementary Materials

The following supporting information can be downloaded at: https://www.mdpi.com/article/10.3390/ijms252011314/s1.
