# Supplementary material for: Transition Metals Coordination by Bis-imidazole-calix[4]arene Ligands with and Without Pyrene Units Grafted at the Large Rim
Source: Int J Mol Sci. 2024 Oct 21;25(20):11314. doi: 10.3390/ijms252011314 (PMC11508328; doi:10.3390/ijms252011314)
Supplement: Supplementary file 1 [file ijms-25-11314-s001.zip › ijms-3236780-supplementary.pdf]

Supplementary Materials

Transition Metals Coordination by Bis-imidazole-calix[4]arene Ligands with and without Pyrene Units Grafted at the Large Rim

Ivana Nikšić-Franjić 1,†, Dijana Pavlović Saftić 2,†, Vilko Smrečki 3, Benoit Colasson 4, Olivia Reinaud 4, Ivo Piantanida 2,* and Aleksandar Višnjevac 1,*

1 Laboratory for Chemical and Biological Crystallography, Division of Physical Chemistry, Ruđer Bošković Institute, Bijenička Cesta 54, 10000 Zagreb, Croatia; ivana.niksic-franjic@irb.hr

2 Laboratory for Biomolecular Interactions and Spectroscopy, Division of Organic Chemistry and Biochemistry, Ruđer Bošković Institute, Bijenička Cesta 54, 10000 Zagreb, Croatia; dija-na.pavlovic.saftic@irb.hr

3 NMR Center, Ruđer Bošković Institute, Bijenička Cesta 54, 10000 Zagreb, Croatia; smrecki@irb.hr

4 Laboratoire de Chimie et de Biochimie Pharmacologiques et Toxicologiques, Université Paris Cité, CNRS, F-75006 Paris, France; benoit.colasson@parisdescartes.fr (B.C.); olivia.reinaud@parisdescartes.fr (O.R.)

* Correspondence: ivo.piantanida@irb.hr (I.P.); aleksandar.visnjevac@irb.hr (A.V.)

† These authors contributed equally to this work.

Contents

1. Synthesis and characterization of ligand **4**. 1
2. X-ray diffraction studies 3
3. NMR studies 4
   3.1. General procedures 4
   3.2. Titrations with Zn salts 4
4. Fluorimetric titrations of compounds **1** - **3** with salts 17
   4.1. Titrations of referent compound **1** with metal salts 17
   4.2. Titrations of pyrene derivatives **2** and **3** with halogenides 22
   4.3. Titrations of pyrene derivatives **2** and **3** with perchlorates 26
   4.4. Titrations of pyrene derivatives **2** and **3** with TMACl 29
5. Fluorescence lifetime measurements 30
6. Computational simulations protocols 35
7. References 37

1. Synthesis and characterization of ligand **4**.

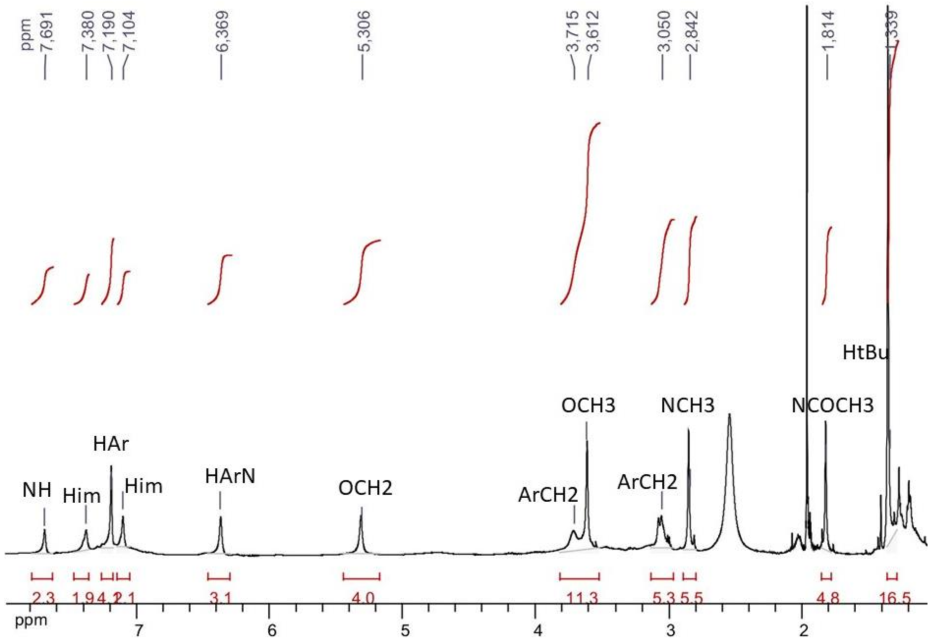

Figure S1. ^{1}H NMR spectra (CD_3CN, 500 MHz, 300 K) of ligand **4** in presence of 1 eq. of $Cu(MeCN)_4PF_6$

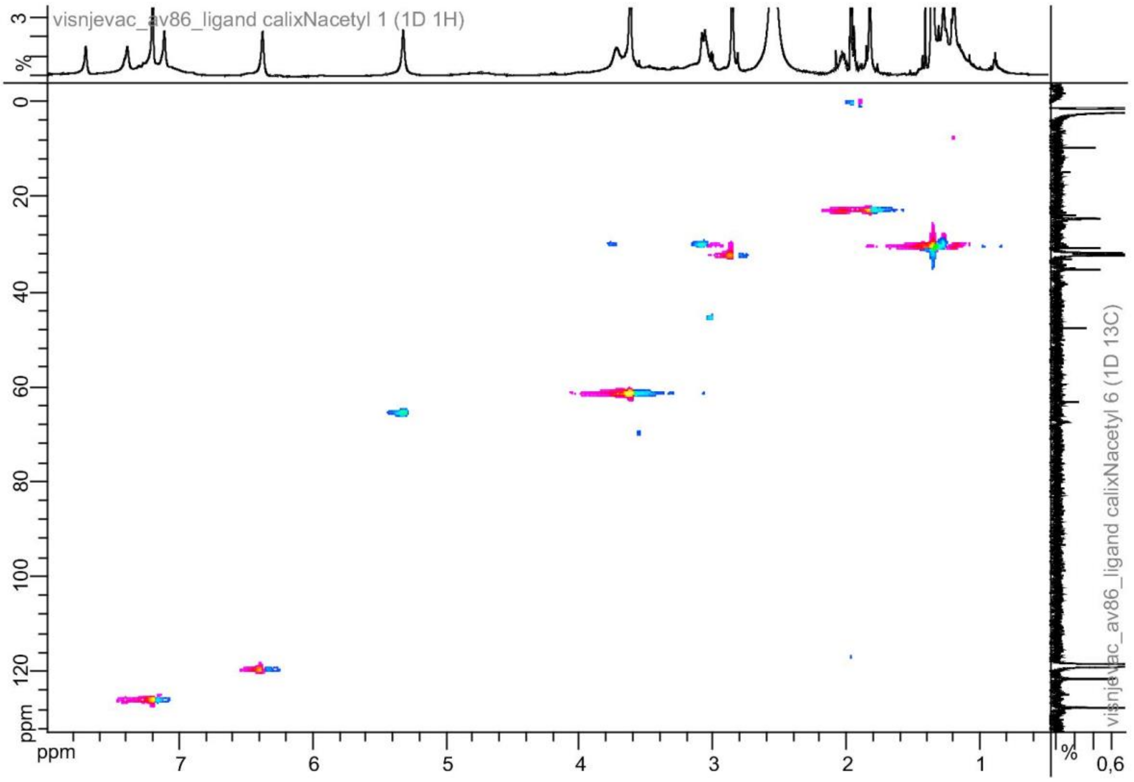

Figure S2. HSQC NMR spectra (CD_3CN, 300 K) of ligand **4** in presence of 1 eq. of $Cu(MeCN)_4PF_6$

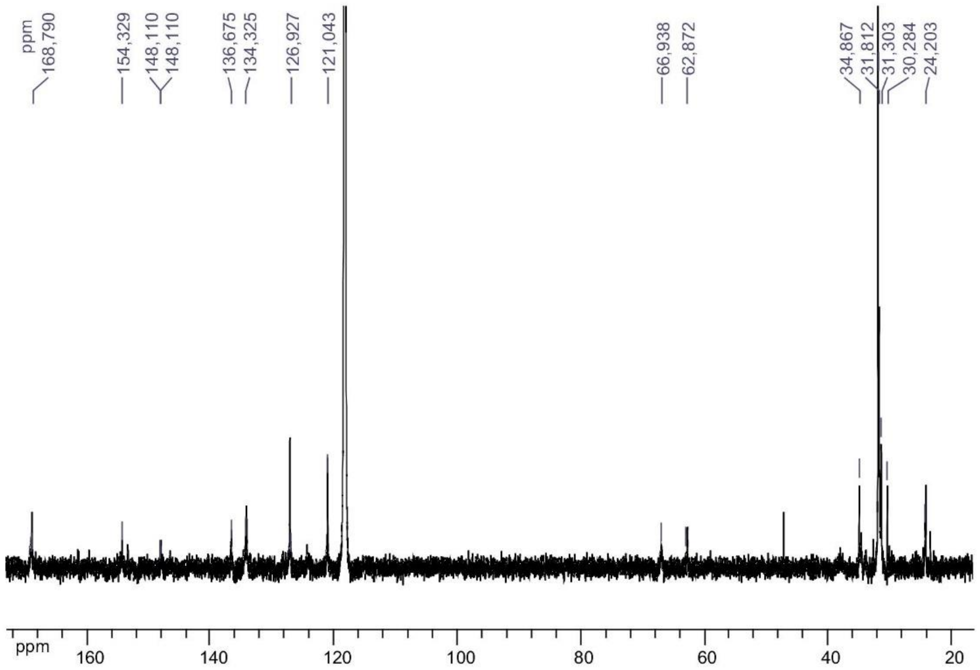

Figure S3. ^{13}C NMR spectra (CD_3CN, 125 MHz, 300 K) of ligand **4** in presence of 1 eq. of $Cu(MeCN)_4PF_6$

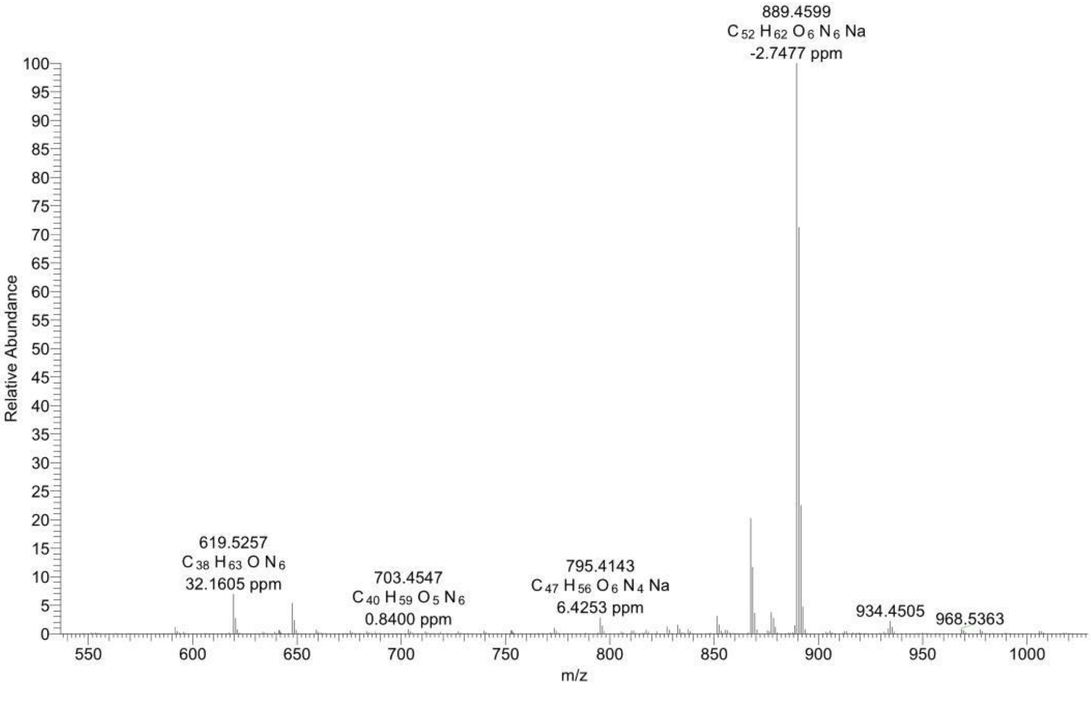

Figure S4. High-resolution mass spectrum (ESI+) of ligand **4**. (**4**: $C_{52}H_{62}N_6NaO_6$, calcd for [**4**+Na]$^+$: 889.4623, found for [**4**+Na]$^+$: 889.4599). (performed on Thermo Scientifc Exactive mass spectrometer)

2. X-ray diffraction studies

Table S1. Crystallographic data

Structure	**1a**	**1b**	**1c**
Brutto form.	$C_{48}H_{54}N_6O_4CuCl_2$	$C_{48}H_{49}N_6O_5Cu$	$C_{98}H_{132}N_{16}$ O_{28} Zn_4
M_r (gmol^{-1})	913.48	853.47	2243.81
Moiety form.	$C_{48}H_{54}N_6O_4CuCl_2$	$C_{48}H_{54}N_6O_5Cu$, $(H_2)O$	C_{96} H_{124} N_{12} O_{14} Zn_4, $2CH_3OH$, $4NO_3$
Crystal color and habit	Yellow prisms	Colourless prisms	Colourless prisms
Cryst. (mm)	0.02 x 0.05 x 0.11	0.15 x 0.18 x 0.20	0.10 x 0.15 x 0.20
F (000)	958	896	1222
μ (mm^{-1})	2.028	0.984	1.668
Space group (No.)	*P*-1 (2)	*P*-1 (2)	*P*-1 (2)
a (Å)	11.4562(9)	11.4501 (2)	11.35818 (18)
b (Å)	14.2526(10)	14.3743 (3)	13.89575 (18)
c (Å)	16.6549(13)	15.7482 (3)	18.4469 (2)
α (°)	110.843(7)	85.607 (2)	97.9838 (10)
β (°)	97.086(7)	81.960 (2)	96.4190 (12)
γ (°)	102.467(6)	78.841 (2)	105.1047 (12)
V (Å^3)	2421.1 (3)	2514.68 (9)	2750.11 (6)
Z (*Z'*)	2 (1)	2 (1)	1 (0.5)
R_{int}	0.0817	0.0910	0.0537
R_σ	0. 1047	0.0741	0.0490
θ_{max} (°)	56.87	79.78	77.48
Unique	6326	10629	10959
Obs. [I>2σ(I)]	4613	8985	9618
Parameters	560	584	707
R_1 [I>2σ(I)]	0.1529	0.1033	0.0405
wR_2, all	0.3859	0.3193	0.1107
S	1.13	1.401	1.067
ρ_{max}, ρ_{min} (eÅ^{-3})	1.81, -0.73, O	1.91, -1.72	0.74, -0.58
Solvent mask	applied	applied	applied

3. NMR studies

3.1. General procedures

NMR spectra were recorded in CD_3CN or MeOD on Bruker AV 600 MHz or Bruker Avance ll 500 spectrometers. Chemical shifts (δ) are expressed in ppm.

3.2. Titrations with Zn salts

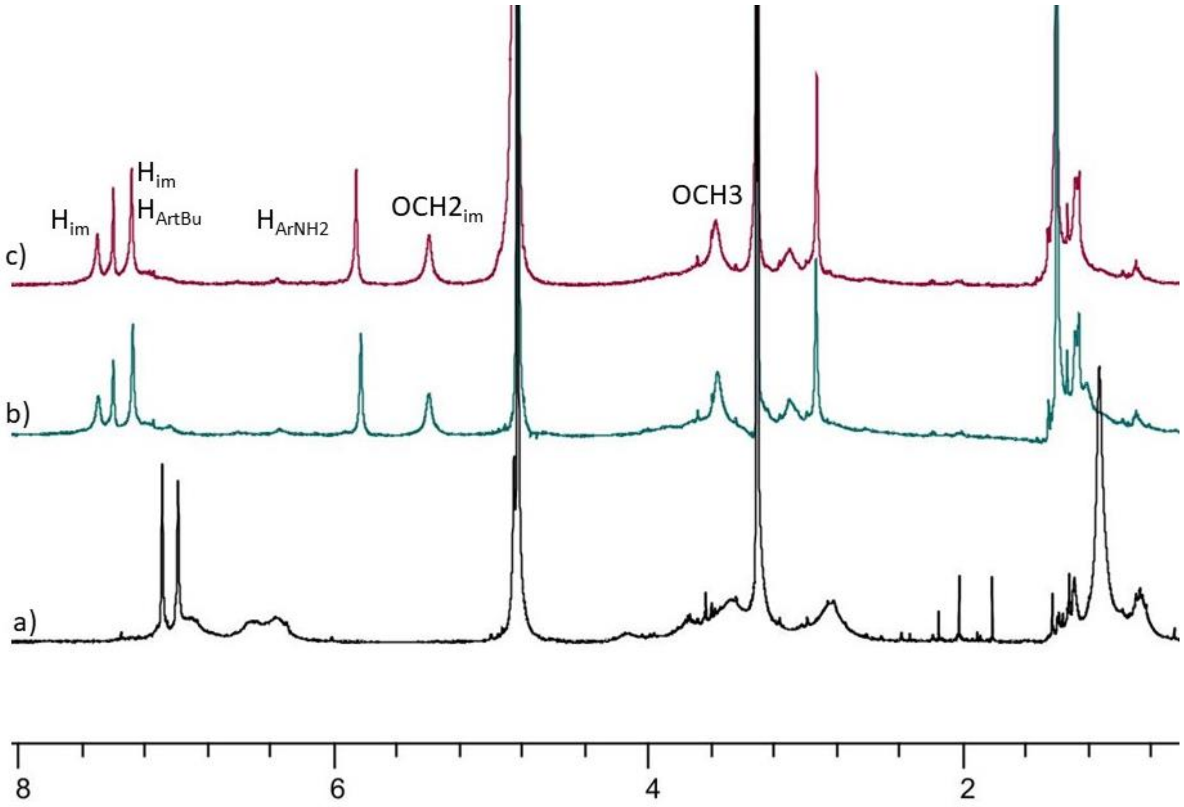

Figure S5. ^1H NMR spectra (CD_3OD, 500 MHz, 300 K) of a) ligand **1**, b) ligand **1** with 1 eq. of $Zn(NO_3)_2$ and c) ligand **1** with 2 eq. of $Zn(NO_3)_2$.

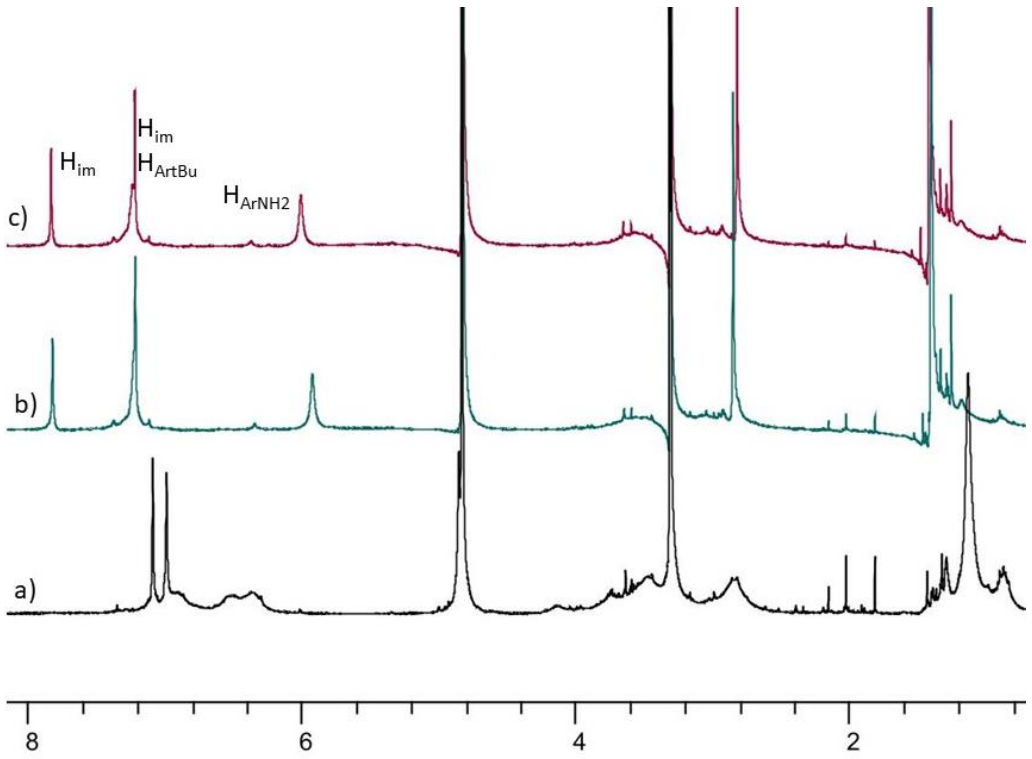

Figure S6. ^{1}H NMR spectra (CD_3OD, 500 MHz, 300 K) of a) ligand **1**, b) ligand **1** with 1 eq. of $ZnCl_2$ and c) ligand **1** with 2 eq. of $ZnCl_2$.

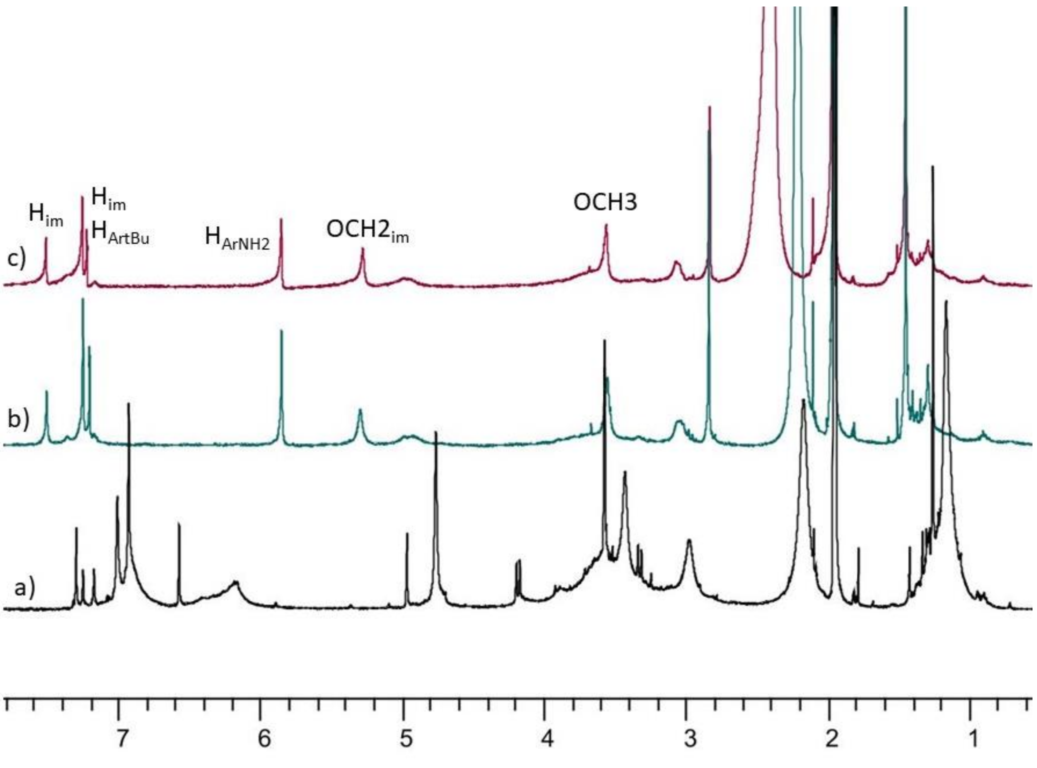

Figure S7. ^{1}H NMR spectra (CD_3CN, 500 MHz, 300 K) of a) ligand **1**, b) ligand **1** with 1 eq. of $Zn(NO_3)_2$ and c) ligand **1** with 2 eq. of $Zn(NO_3)_2$.

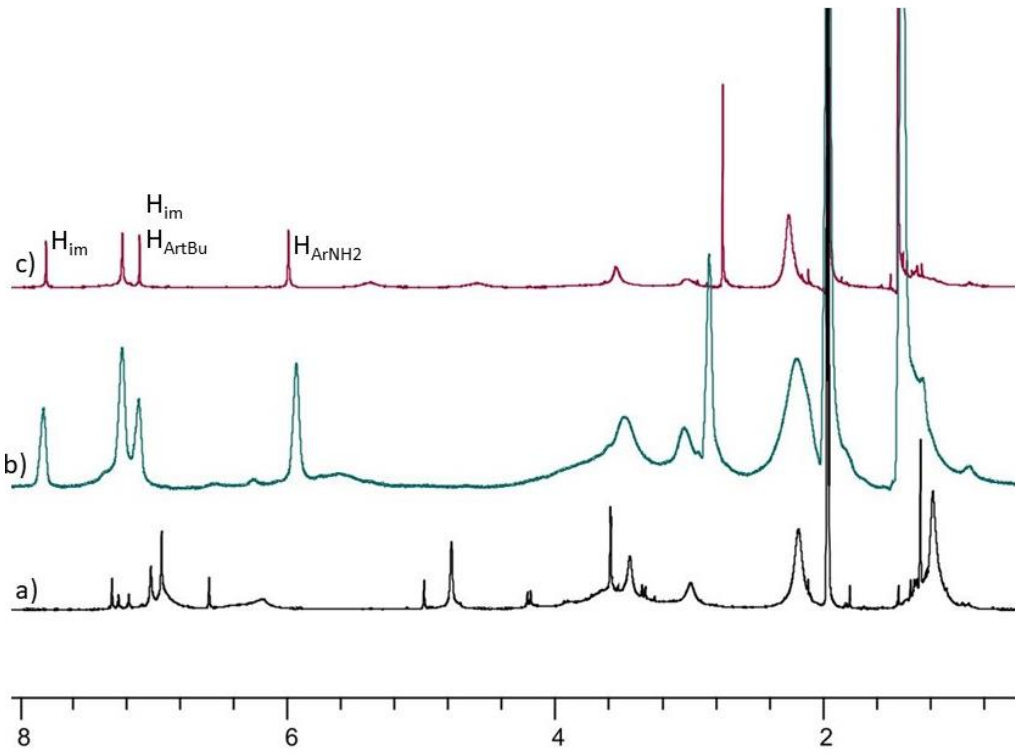

Figure S8. ^{1}H NMR spectra (CD_3CN, 500 MHz, 300 K) of a) ligand **1**, b) ligand **1** with 1 eq. of $ZnCl_2$ and c) ligand **1** with 2 eq. of $ZnCl_2$.

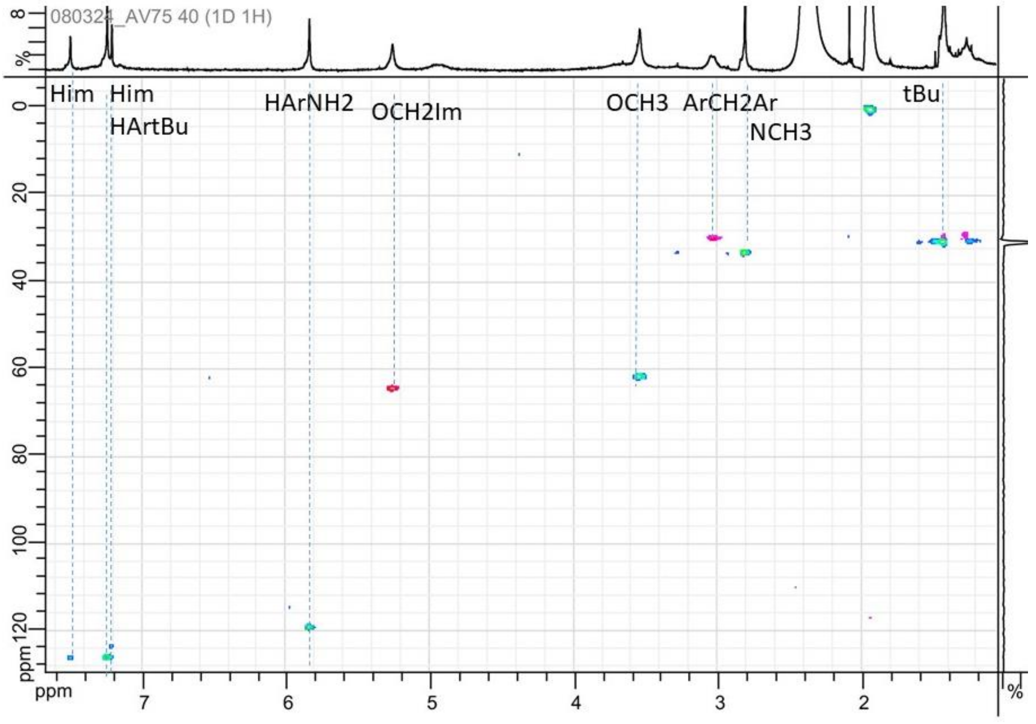

Figure S9. HSQC NMR spectra (CD_3CN, 500 MHz, 300 K) of ligand **1** with 2 eq. of $Zn(NO_3)_2$.

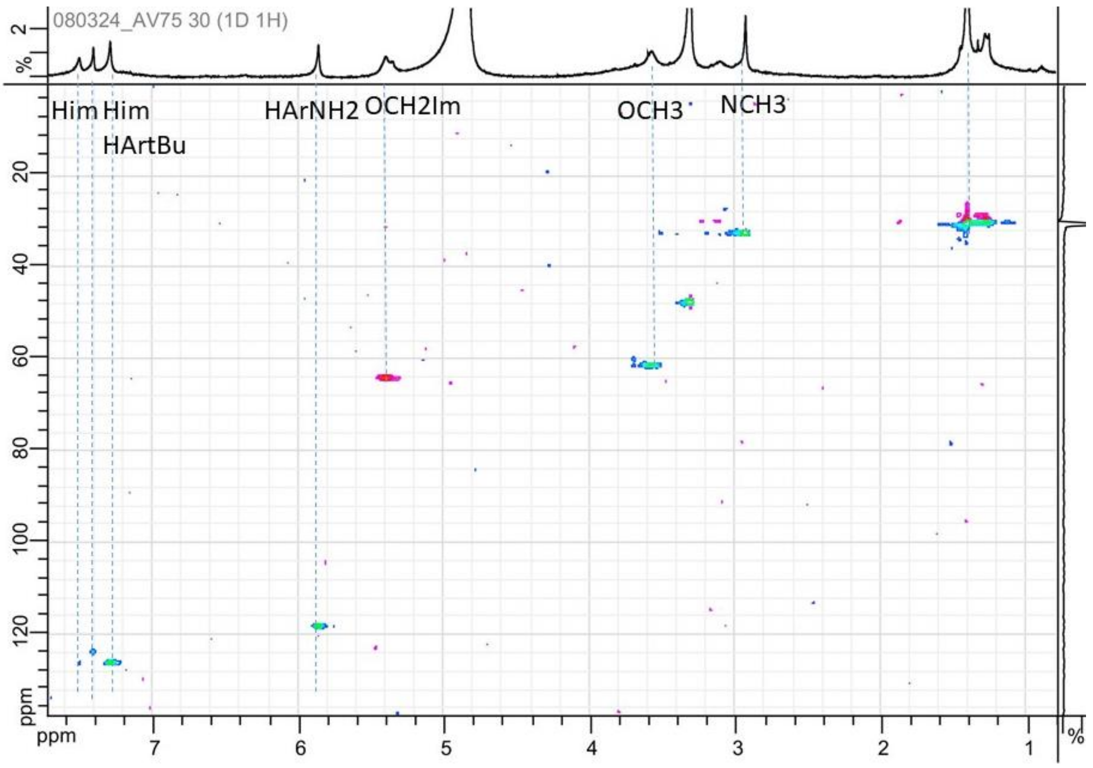

Figure S10. HSQC NMR spectra (CD_3OD, 500 MHz, 300 K) of ligand **1** with 2 eq. of $Zn(NO_3)_2$.

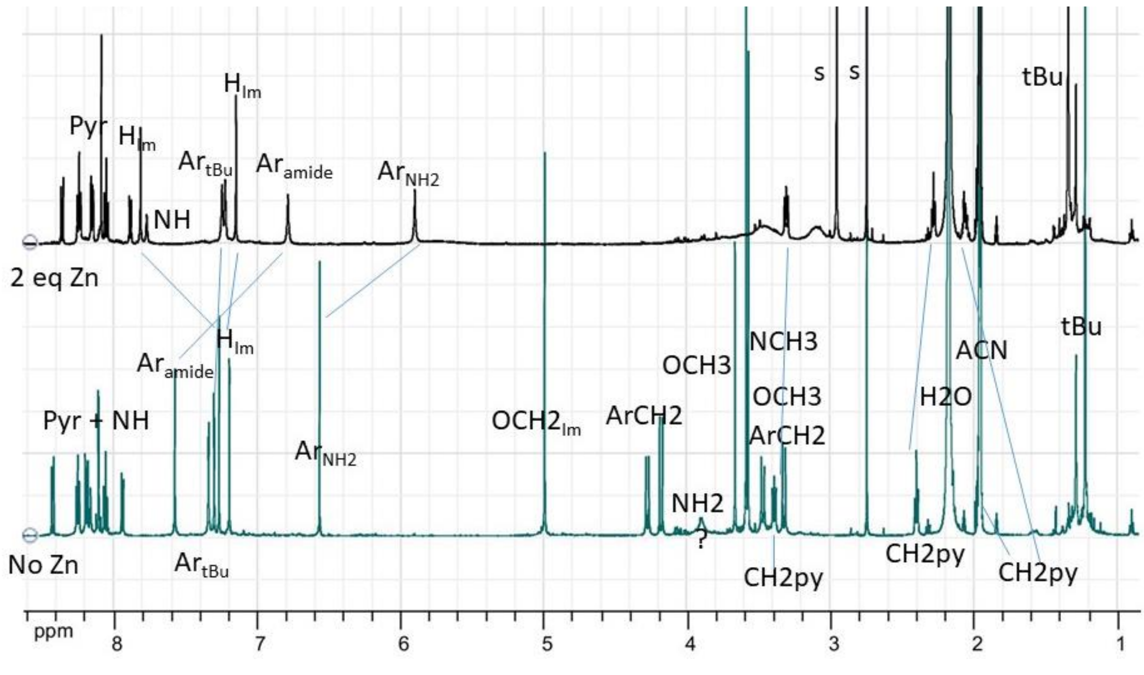

Figure S11. ^{1}H NMR spectra (CD_3CN, 600 MHz, 300 K) of a) **2**and b) **2** with 2 eq. of $ZnCl_2$.

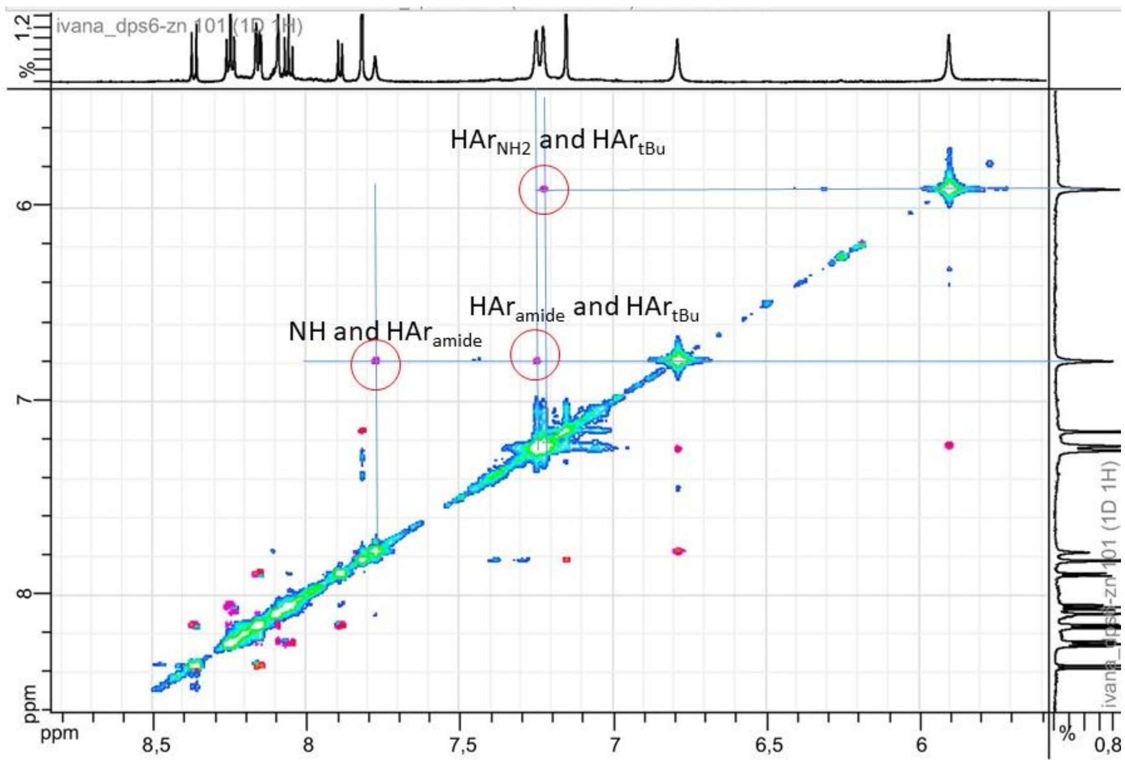

Figure S12. Partial NOESY NMR spectrum (CD_3CN, 600 MHz, 300 K) of **2** with 2 eq of $ZnCl_2$.

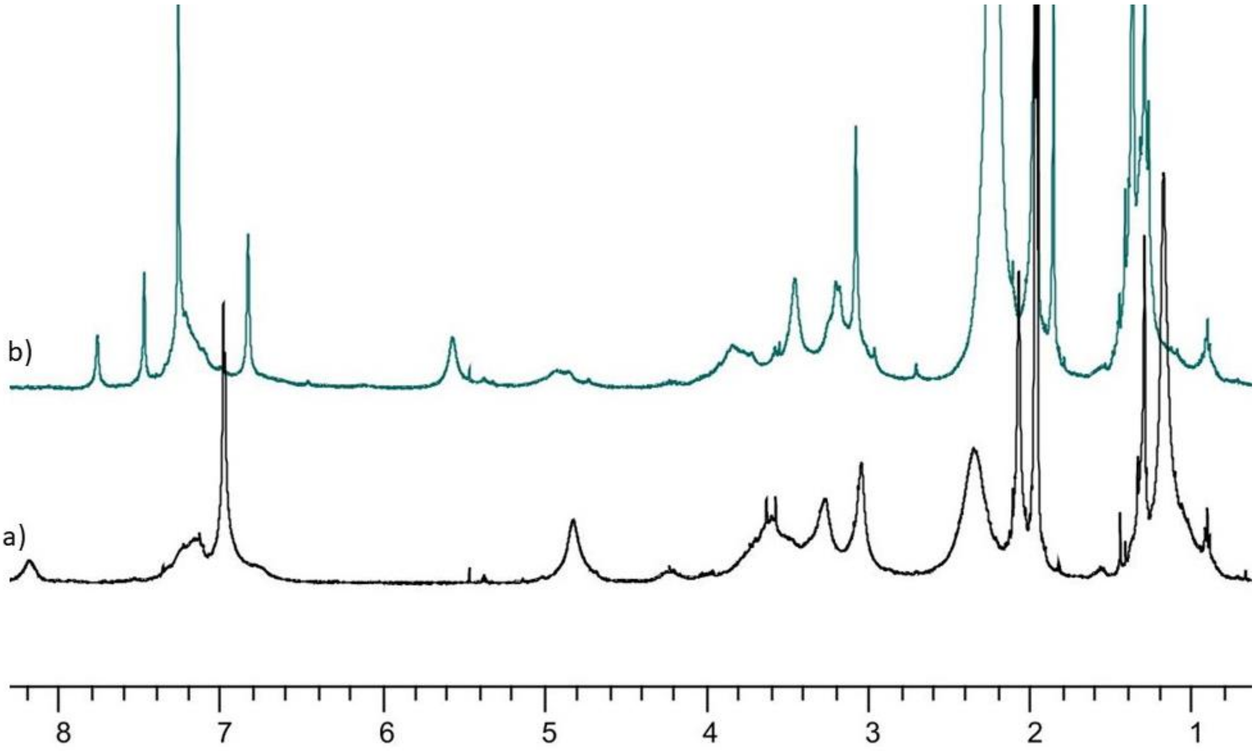

Figure S13. ^{1}H NMR spectra (CD_3CN, 500 MHz, 300 K) of a) ligand **4** and b) ligand **4** with 0.5 eq. of $Zn(NO_3)_2$.

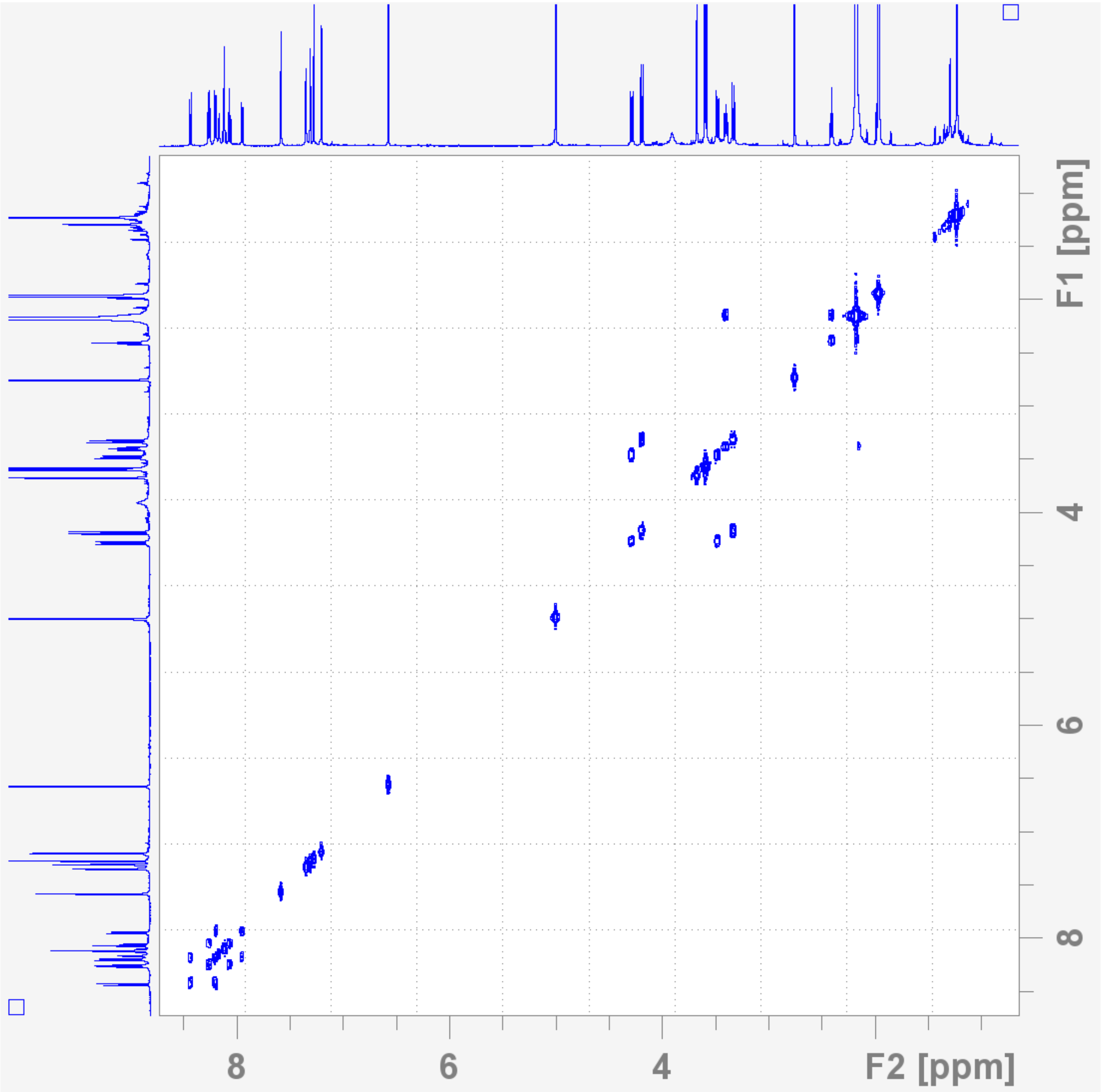

Figure S14. COSY spectrum of **2** (1.97×10^{-3} M, CD_3CN, PABBO, 300 K).

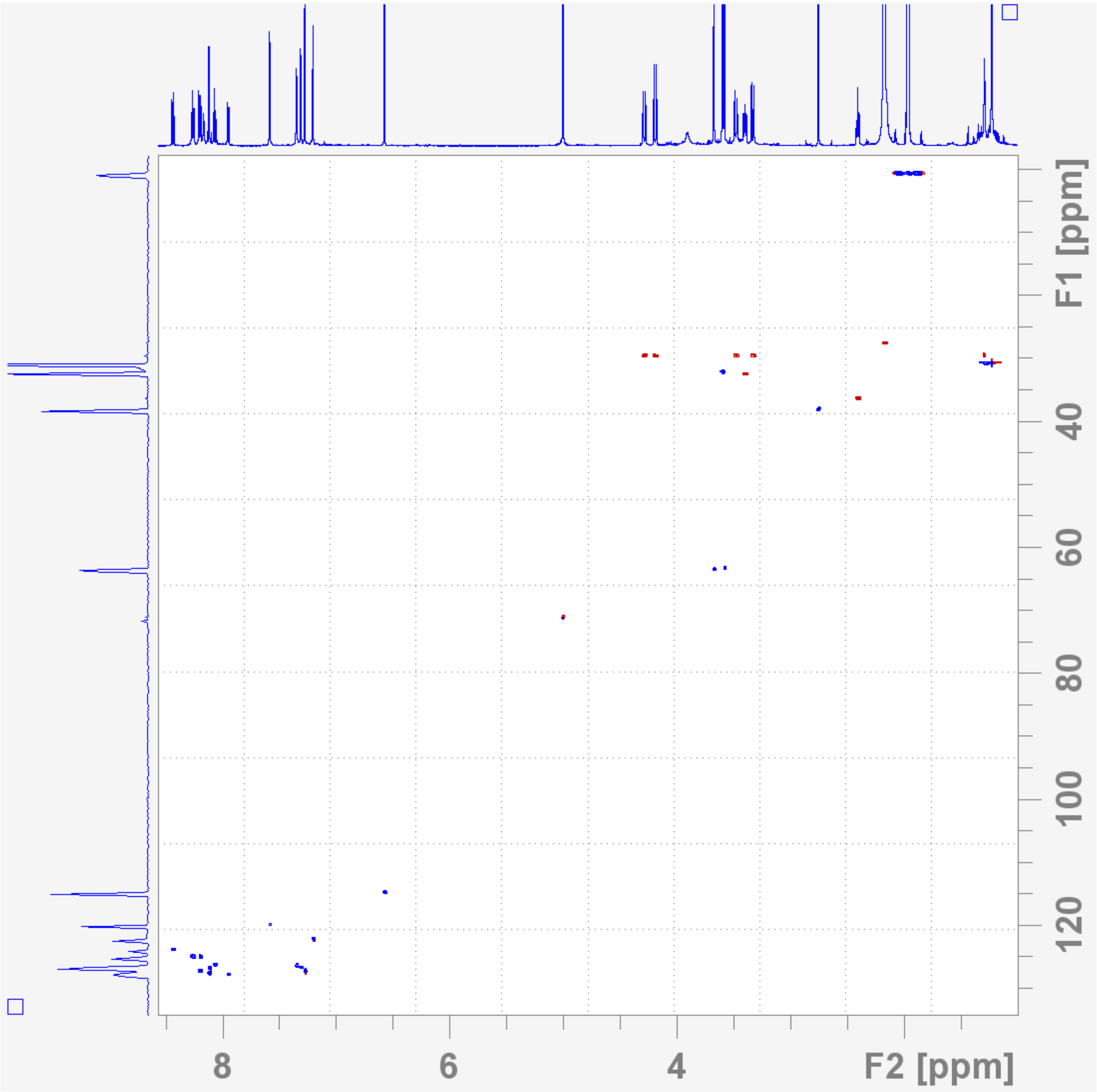

Figure S15. HSQC spectrum of **2** (1.97×10^{-3} M, CD_3CN, PABBO, 300 K).

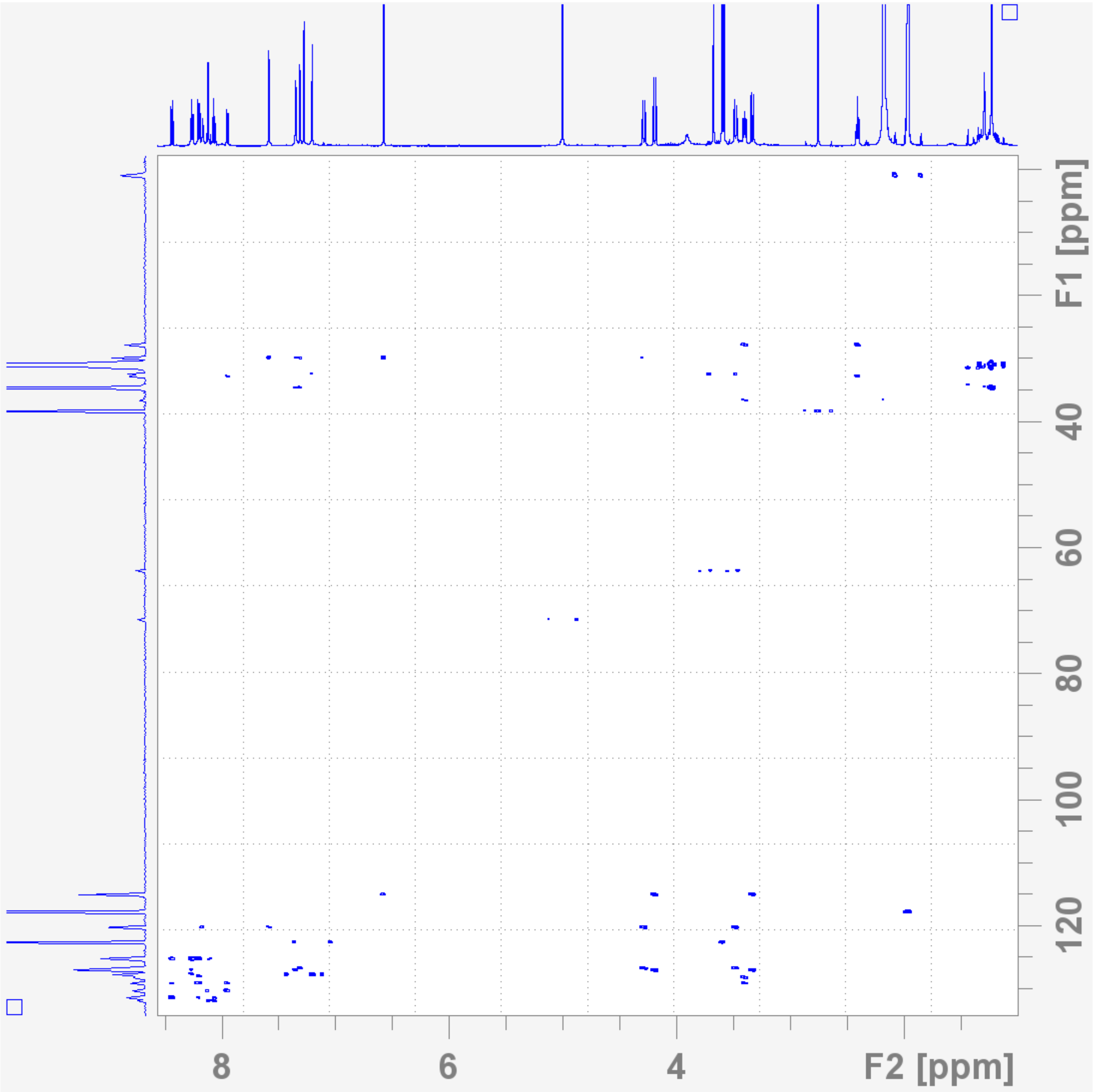

Figure S16. HMBC spectrum of 2 (1.6×10^{-3} M, CD_3CN, PABBO, 300 K).

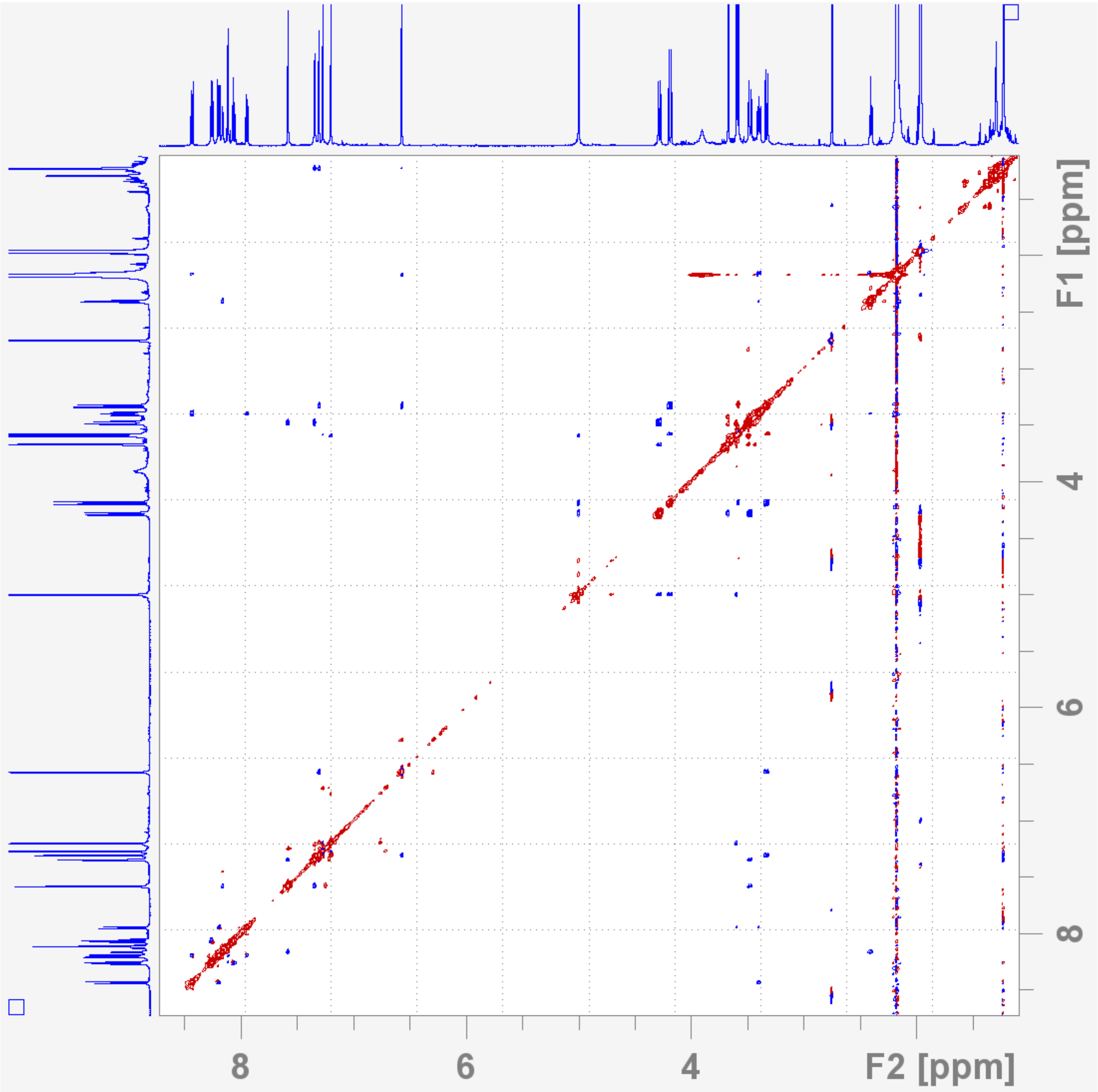

Figure S17. NOESY spectrum of **2** (1.97×10^{-3} M, CD_3CN, PABBO, 300 K).

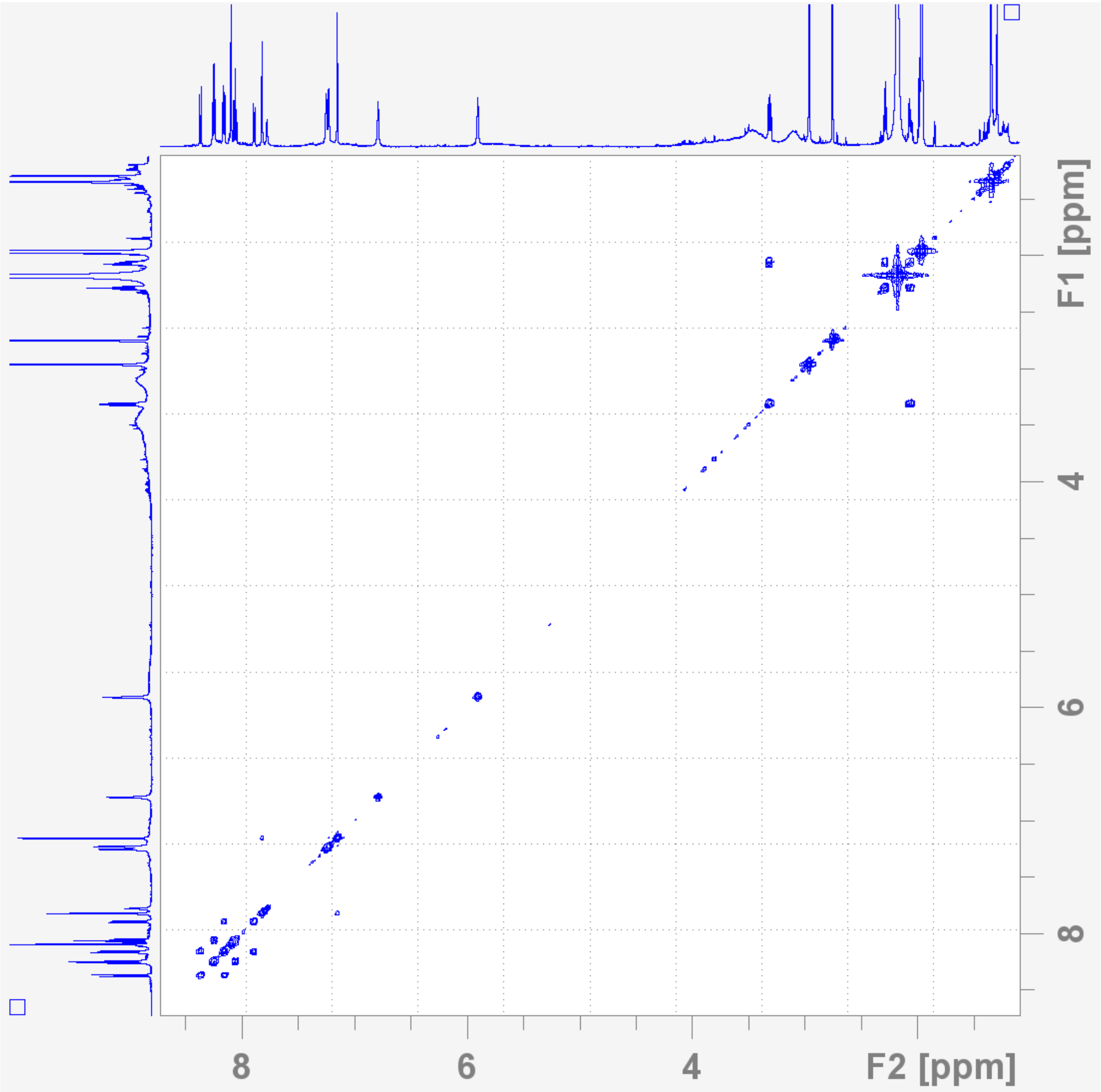

Figure S18. COSY spectrum of the Zn(II) complex of **2** (550 µL 1.97 × 10^{-3} M of **2** + 198.2 µL 9.94 × 10^{-3} M of $ZnCl_2$, CD_3CN, 300 K).

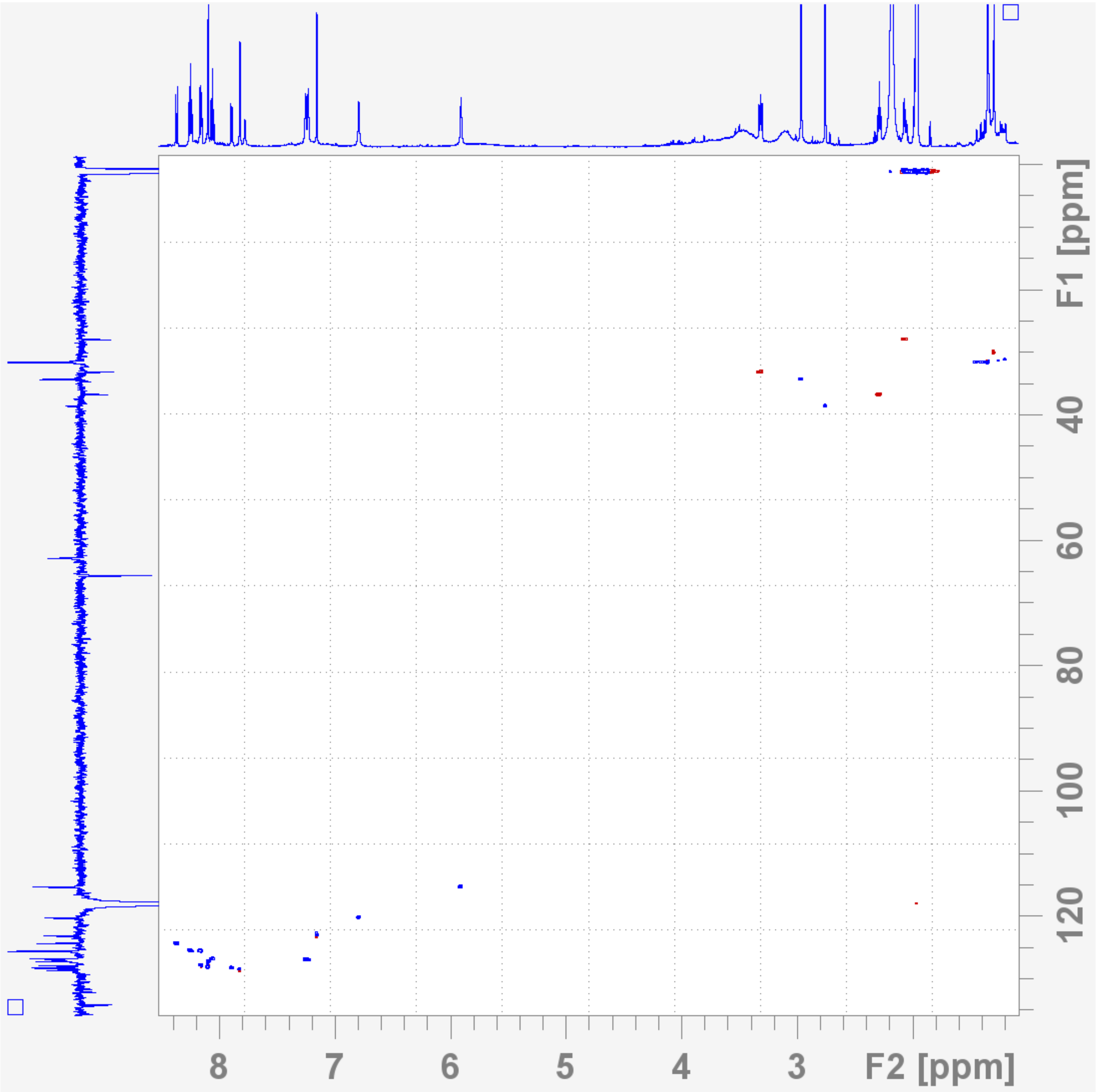

Figure S19. HSQC spectrum of the Zn(II) complex of **2** (550 µL 1.97 × 10^{-3} M of **2** + 198.2 µL 9.94 × 10^{-3} M of $ZnCl_2$, CD_3CN, PABBO, 300 K).

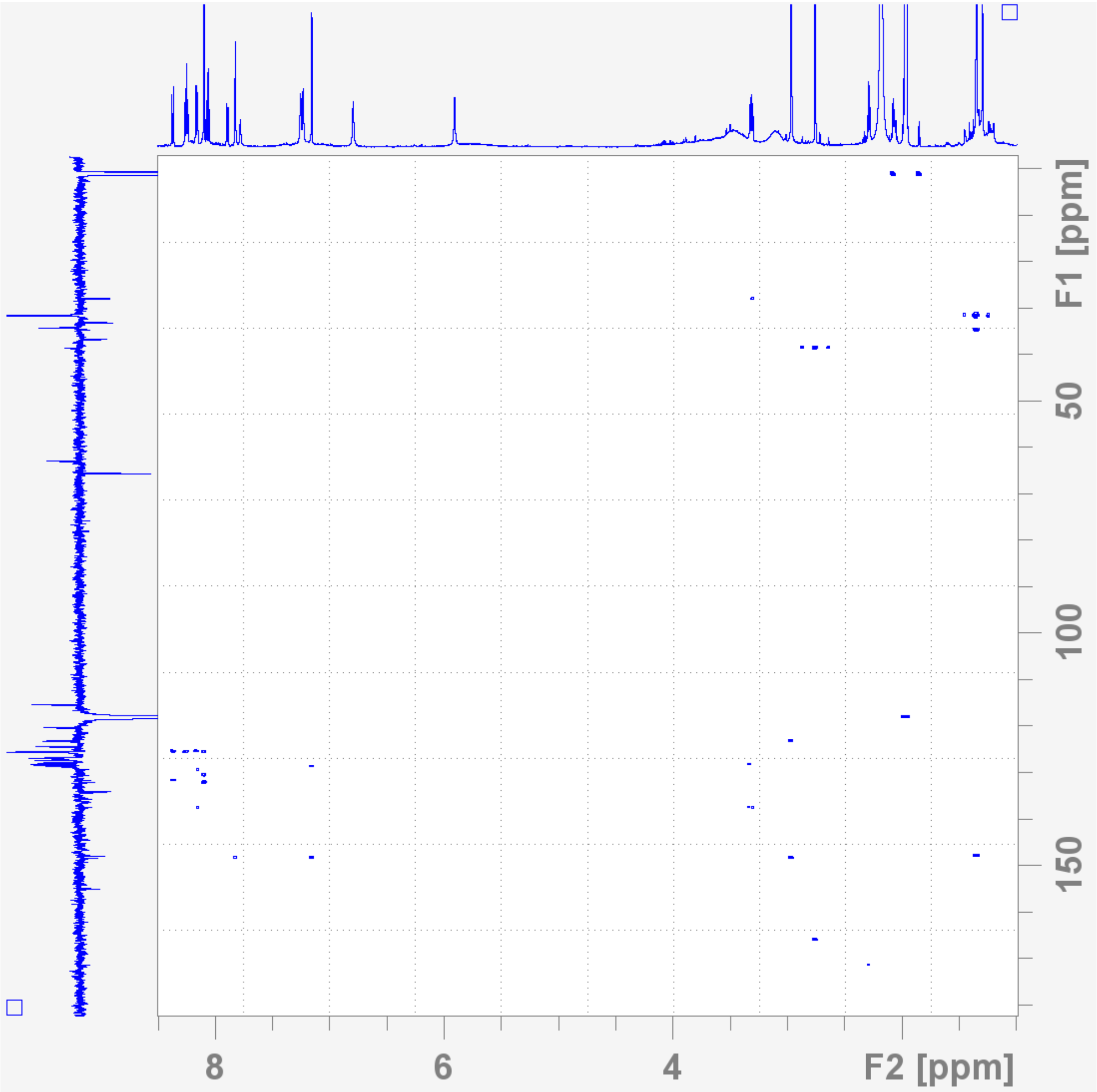

Figure S20. HMBC spectrum of the Zn(II) complex of **2** (550 µL 1.97×10^{-3} M of **2** + 198.2 µL 9.94×10^{-3} M of $ZnCl_2$, CD_3CN, PABBO, 300 K).

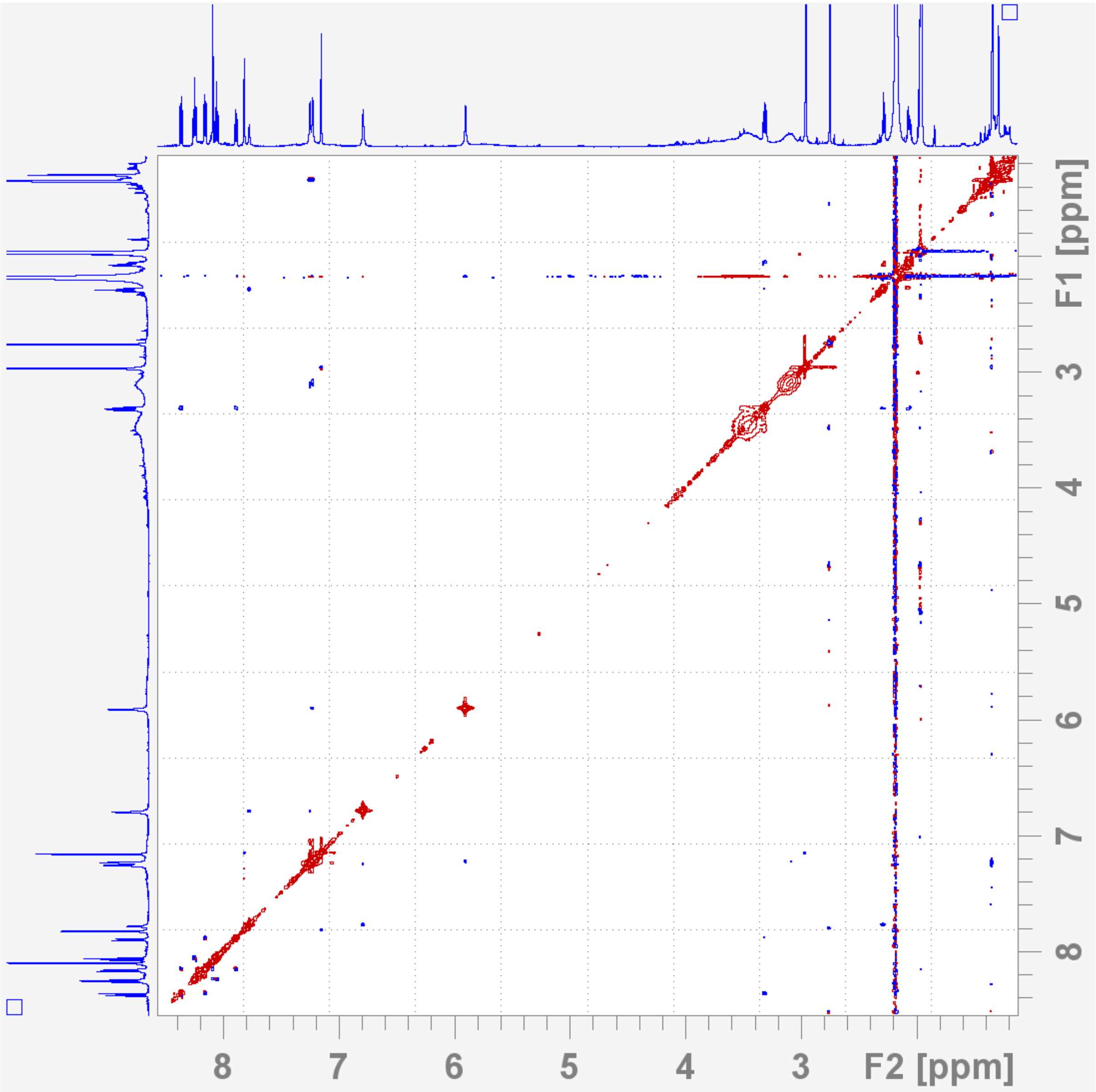

Figure S21. NOESY spectrum of the Zn(II) complex of **2** (550 µL 1.97×10^{-3} M of **2** + 198.2 µL 9.94×10^{-3} M of $ZnCl_2$, CD_3CN, PABBO, 300 K).

4. Fluorimetric titrations of compounds **1** - **3** with salts

4.1. Titrations of referent compound **1** with metal salts

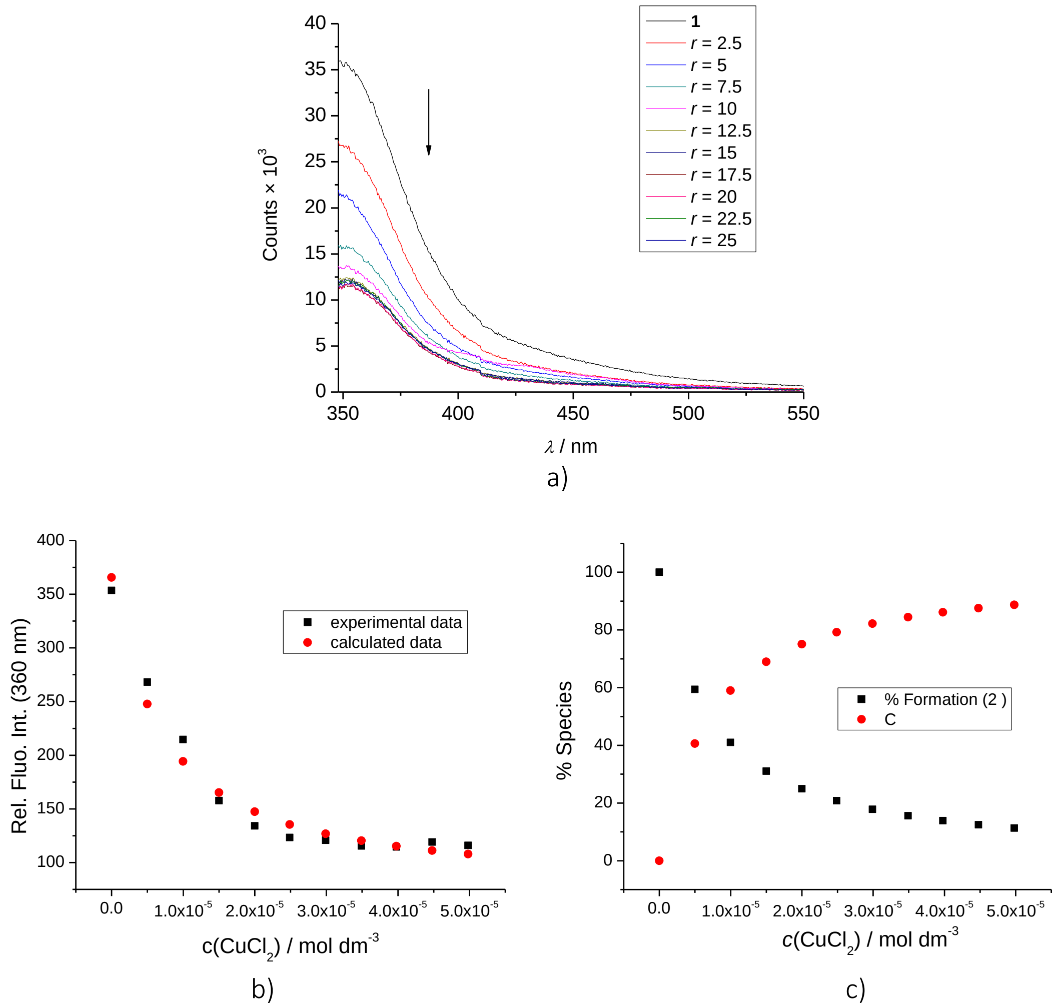

Figure S22. a) Fluorimetric titration of **1** (c = 2 × 10^{-6} mol dm^{-3}; λ_{exc} = 300 nm) with $CuCl_2$ (c = 1 × 10^{-2} mol dm^{-3}), b) dependence of fluorescence at λ_{max} = 360 nm on $c(CuCl_2)$ and agreement between experimental and calculated data at 360 nm; c) the percentage of chromophore species in solution in dependence to c(metal cation). Titration data are processed by using the Specfit program. Done in water; r = $[CuCl_2]$ / [**1**].

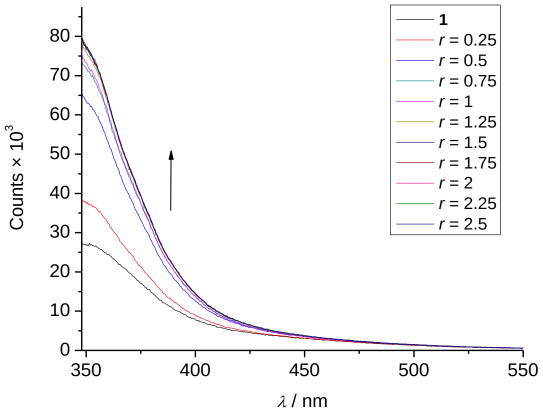

a)

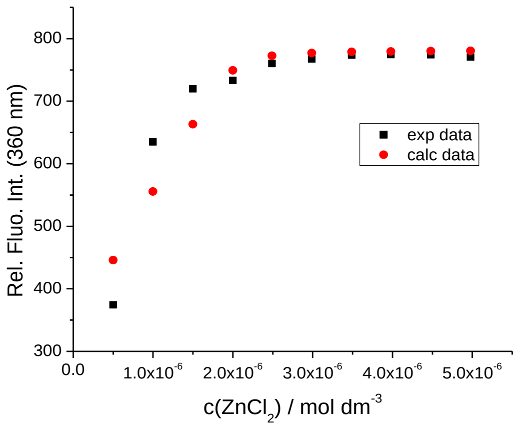

b)

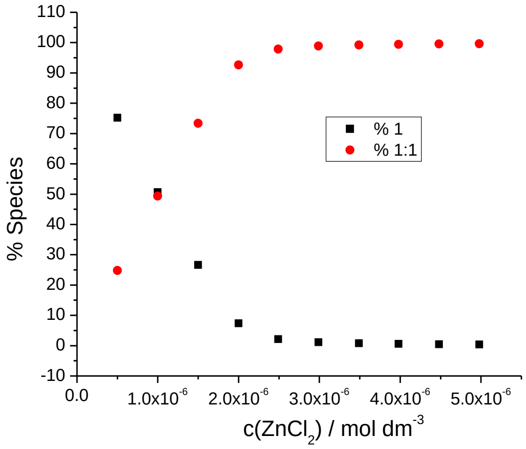

c)

Figure S23. a) Fluorimetric titration of **1** ($c = 2 \times 10^{-6}$ mol dm^{-3}; $\lambda_{exc} = 300$ nm) with $ZnCl_2$ ($c = 1 \times 10^{-3}$ mol dm^{-3}), b) dependence of fluorescence at λ_{max} = 360 nm on $c(ZnCl_2)$ and agreement between experimental and calculated data at 360 nm; c) the percentage of chromophore species in solution in dependence to *c*(metal cation). Titration data are processed by using the Specfit program. Done in water; $r = [ZnCl_2] / [\mathbf{1}]$.

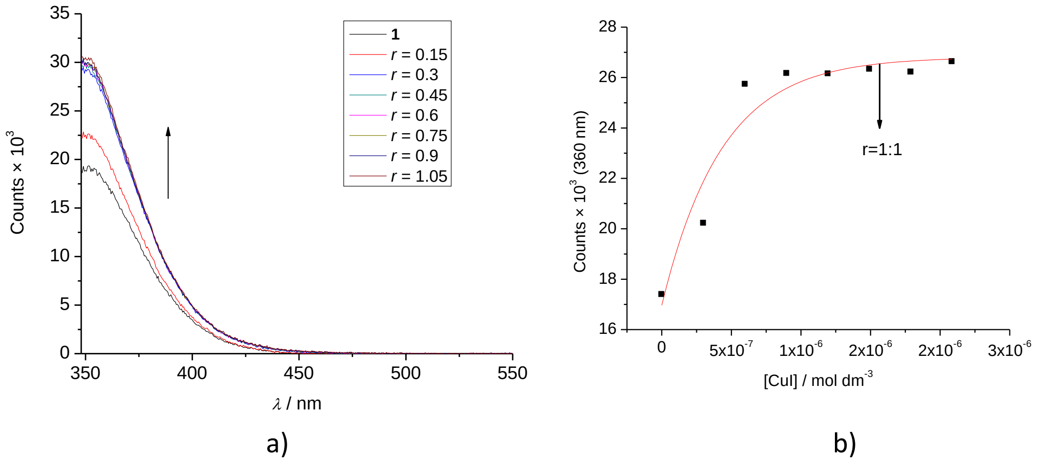

Figure S24. a) Fluorimetric titration of **1** ($c = 2 \times 10^{-6}$ mol dm^{-3}; $\lambda_{exc} = 300$ nm) with CuI ($c = 3 \times 10^{-4}$ mol dm^{-3}), and b) dependence of fluorescence at $\lambda_{max} = 360$ nm on c(CuI). The linear emission increase proportional to c(metal cation) up to ratio [dye] / [metal cation] = 1:1 strongly supports log $K > 8$. Done in water; r = [CuI] / [**1**].

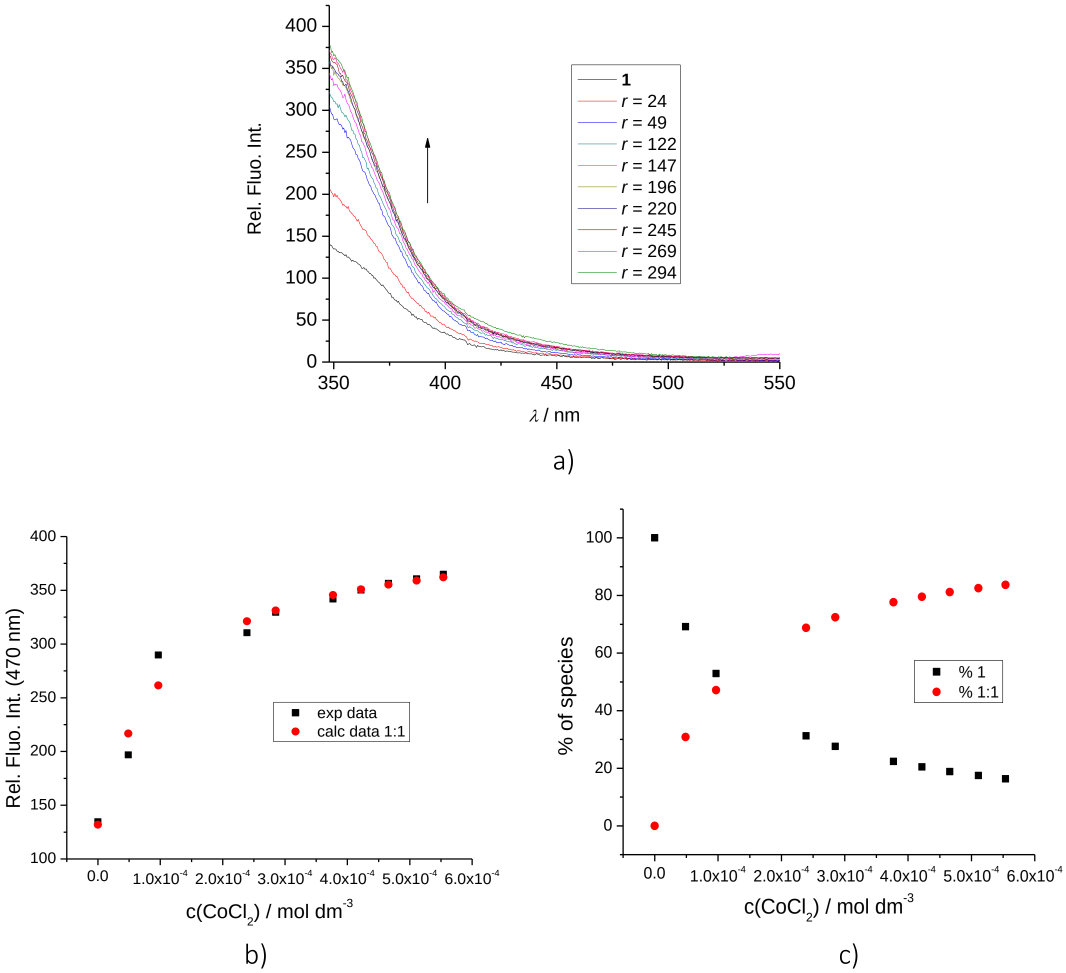

Figure S25. a) Fluorimetric titration of **1** ($c = 2 \times 10^{-6}$ mol dm^{-3}; $\lambda_{exc} = 300$ nm) with $CoCl_2 \times 6\ H_2O$ ($c = 9.8 \times 10^{-3}$ mol dm^{-3}), b) dependence of fluorescence at $\lambda_{max} = 360$ nm on $c(CoCl_2 \times 6\ H_2O)$ and agreement between experimental and calculated data at 360 nm; c) the percentage of chromophore species in solution in dependence to c(metal cation). Titration data are processed by using the Specfit program. Done in water; $r = [CoCl_2 \times 6\ H_2O] / [\mathbf{1}]$.

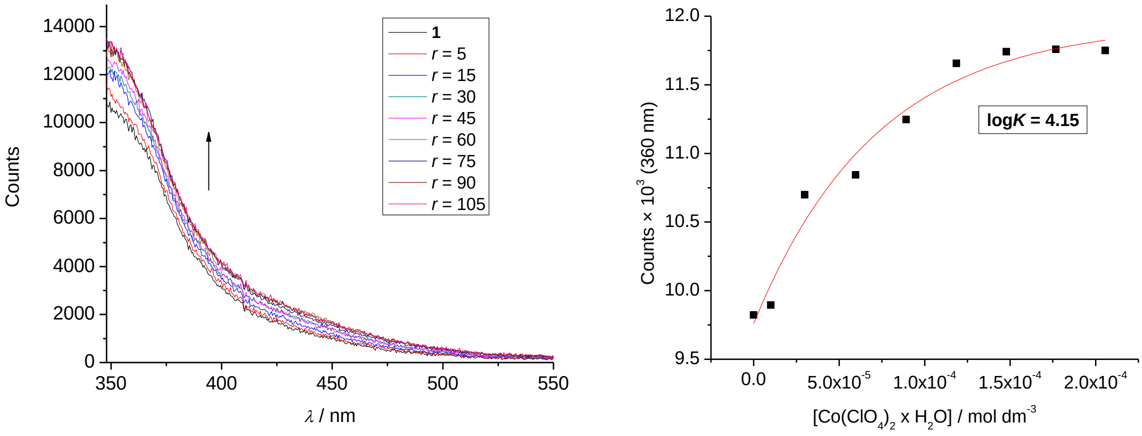

a) b)

Figure S26. a) Fluorimetric titration of **1** ($c = 2 \times 10^{-6}$ mol dm^{-3}; $\lambda_{exc} = 300$ nm) with $Cd(ClO_4)_2 \times H_2O$ ($c = 1 \times 10^{-2}$ mol dm^{-3}), b) dependence of fluorescence at $\lambda_{max} = 360$ nm on $c(Cd(ClO_4)_2 \times H_2O)$. Titration data are processed by using the Origin 7.5 program. Done in water; $r = [Cd(ClO_4)_2 \times H_2O] / [\mathbf{1}]$.

4.2. Titrations of pyrene derivatives **2** and **3** with halogenides

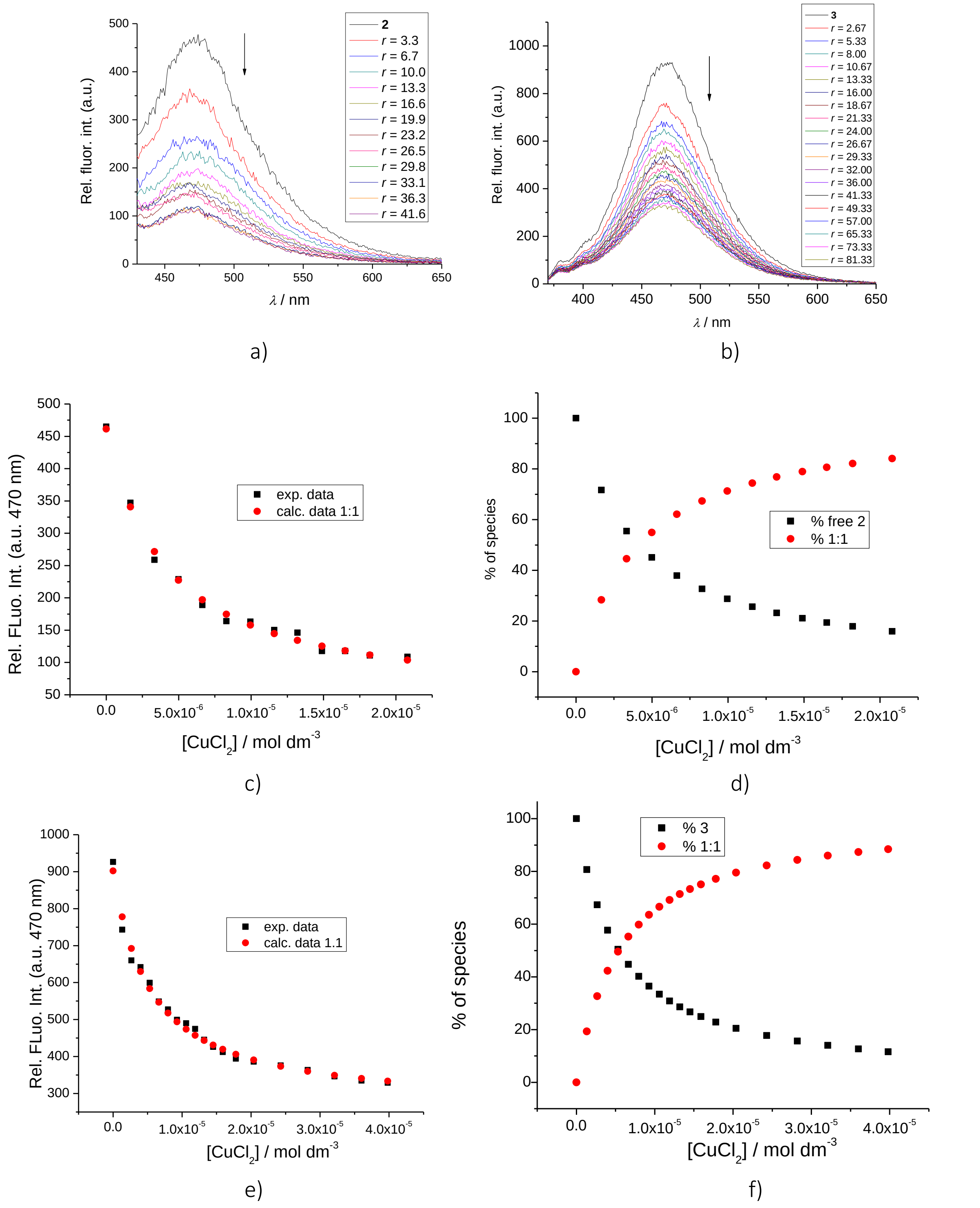

Figure S27. Fluorimetric titration of: a) **2** and b) **3** (c(**dye**) = 5 × 10^{-7} mol dm^{-3}; λ_{exc}= 350 nm) with $CuCl_2$ (c = 2 × 10^{-3} mol dm^{-3}); c,e) agreement between experimental and calculated data at 470 nm; d,f) percentage of chromophore species in solution in dependence to c(metal cation). Titration data are processed by using the Specfit program. Done in water; r = [$CuCl_2$] / [compound].

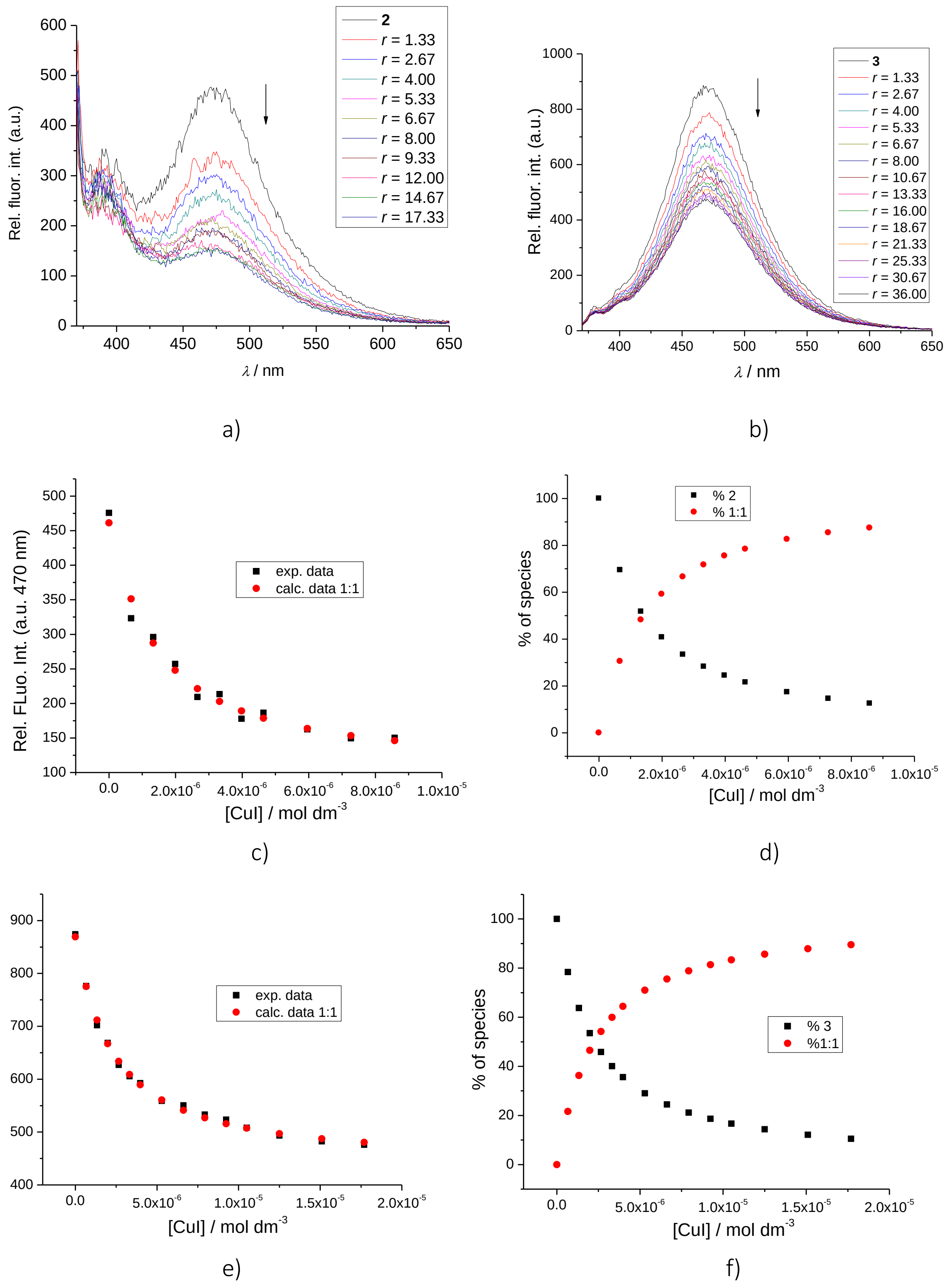

Figure S28. Fluorimetric titration of: a) **2** and b) **3** (c(**dye**) = 5 × 10^{-7} mol dm^{-3}; λ_{exc}= 350 nm) with CuI (c = 1 × 10^{-3} mol dm^{-3}); c,e) agreement between experimental and calculated data at 470 nm; d,f) percentage of chromophore species in solution in dependence to c(metal cation). Titration data are processed by using the Specfit program. Done in water; r = [CuI] / [compound].

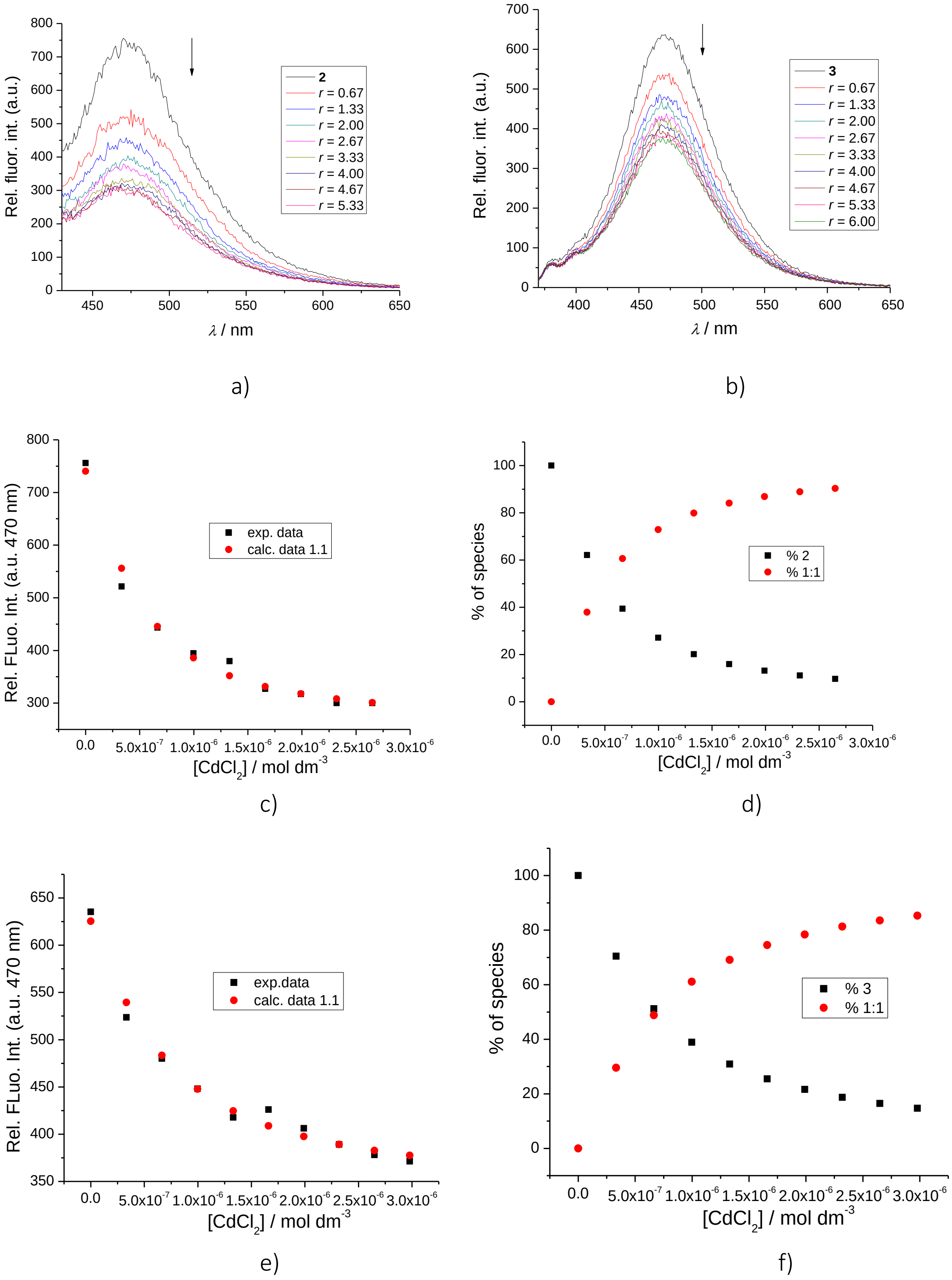

Figure S29. Fluorimetric titration of: a) **2** and b) **3** (c(**dye**) = 5 × 10^{-7} mol dm^{-3}; λ_{exc}= 350 nm) with $CdCl_2$ × 2.5 H_2O (c = 5 × 10^{-4} mol dm^{-3}); c,e) agreement between experimental and calculated data at 470 nm; d,f) percentage of chromophore species in solution in dependence to c(metal cation). Titration data are processed by using the Specfit program. Done in water; r = [$CdCl_2$ × 2.5 H_2O] / [compound].

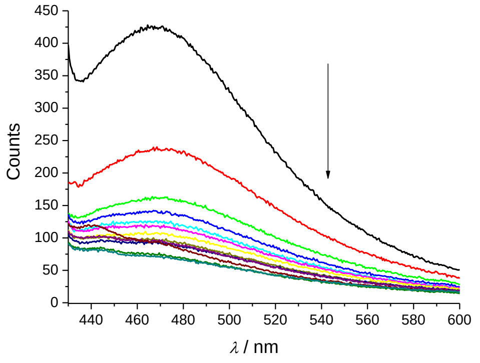

a)

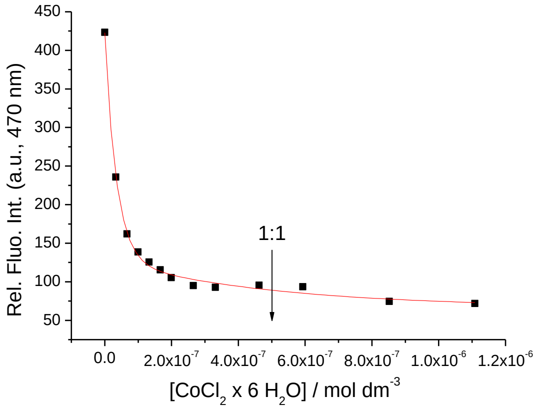

b)

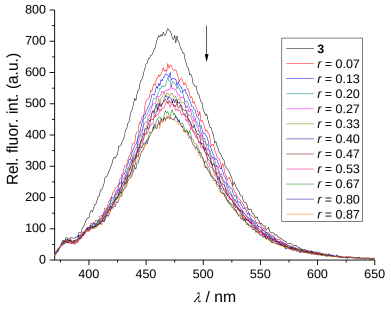

c)

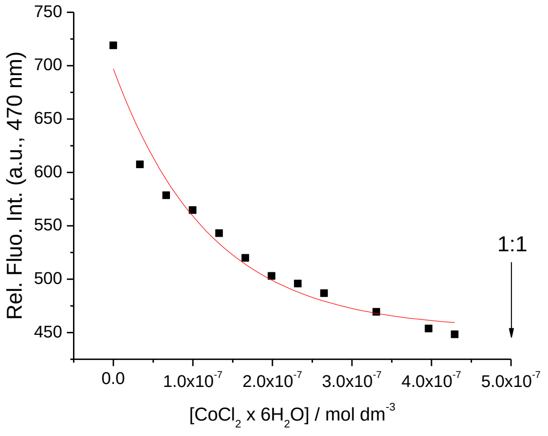

d)

Figure S30. Fluorimetric titration of: a) **2** and c) **3** (c) (c(**dye**) = 5 × 10^{-7} mol dm^{-3}; λ_{exc}= 350 nm) with $CoCl_2$ × 6 H_2O (c = 5 × 10^{-5} mol dm^{-3}), and agreement between experimental and calculated data at 470 nm for **2** (b) and **3** (d). Titration data are processed by using the Specfit program. Done in water; r = [$CoCl_2$ × 6 H_2O] / [compound]. The end of both titrations at ratio [dye] / [metal cation] = 1:1 strongly supports log K > 8.

4.3. Titrations of pyrene derivatives **2** and **3** with perchlorates

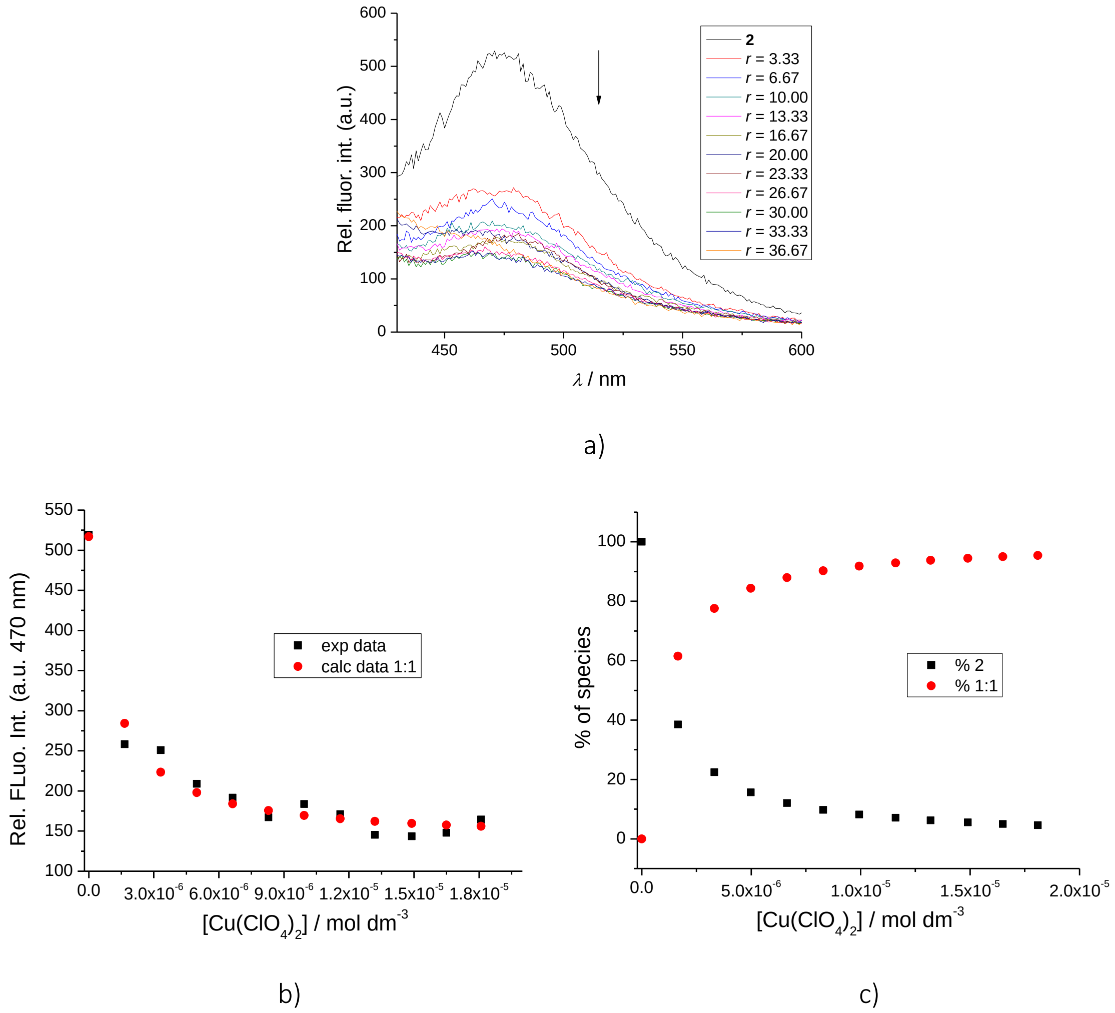

Figure S31. Fluorimetric titration of: a) **2** ($c = 5 \times 10^{-7}$ mol dm^{-3}; λ_{exc}= 350 nm) with $Cu(ClO_4)_2 \times 6\ H_2O$ ($c = 2 \times 10^{-3}$ mol dm^{-3}); b) agreement between experimental and calculated data at 360 nm; c) percentage of chromophore species in solution in dependence to c(metal cation). Titration data are processed by using the Specfit program. Done in water; r = [$Cu(ClO_4)_2 \times 6\ H_2O$] / [compound].

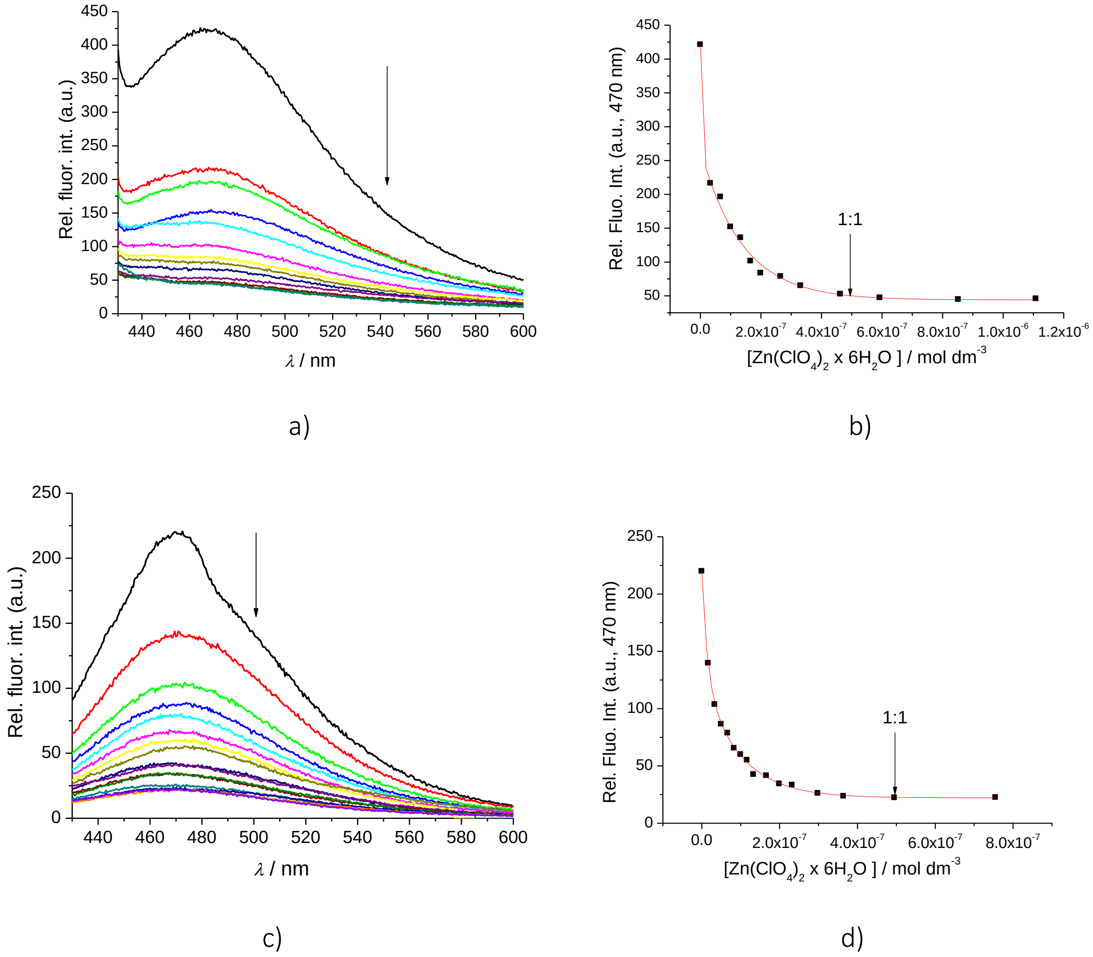

Figure S32. Fluorimetric titration of: a) **2** (c = 1 × 10^{-6} mol dm^{-3}; λ_{exc}= 350 nm) and c) **3** (c) (c = 5 × 10^{-7} mol dm^{-3}; λ_{exc}= 350 nm) with $Zn(ClO_4)_2$ × 6 H_2O (c = 5 × 10^{-5} mol dm^{-3}) and agreement between experimental and calculated data at 470 nm for **2** (b) and **3** (d). Titration data are processed by using the Specfit program. Done in water. The end of both titrations at ratio [dye] / [metal cation] = 1:1 strongly supports log K > 8.

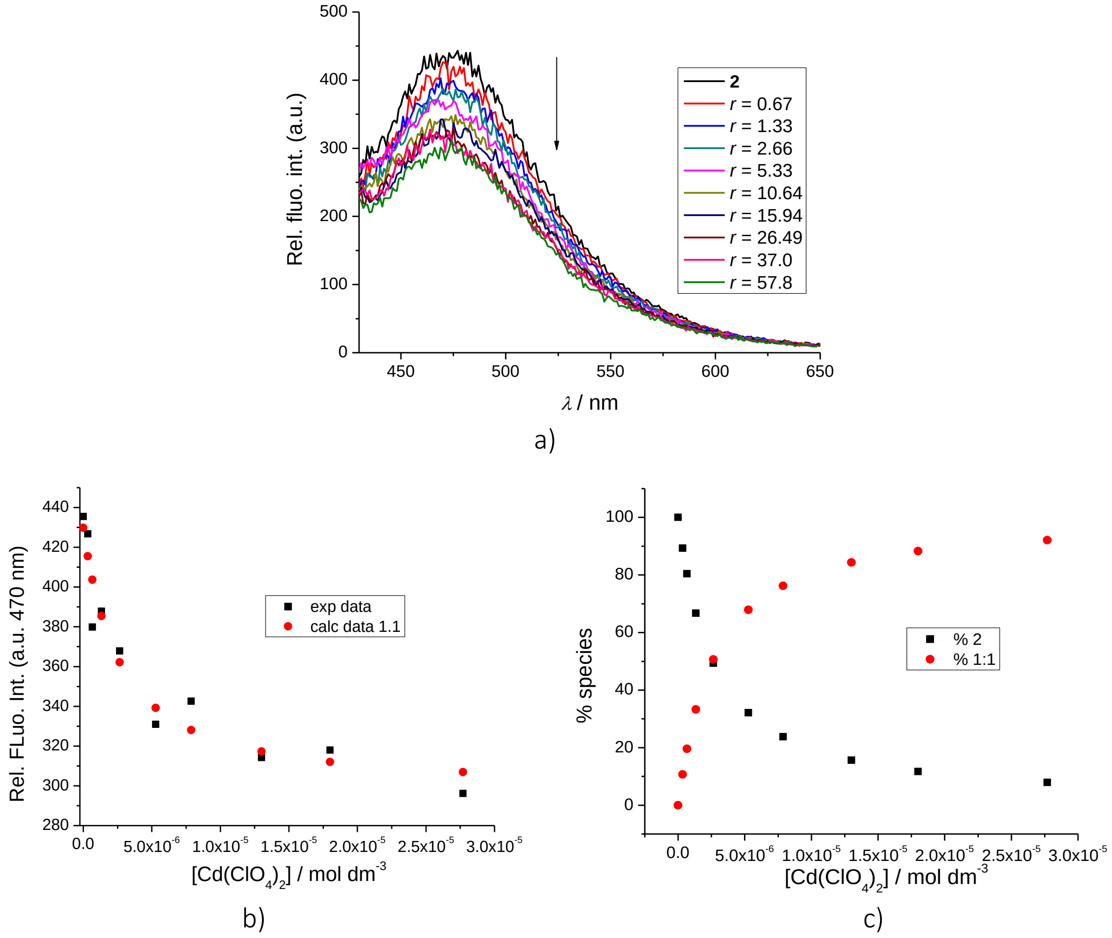

Figure S33. Fluorimetric titration of: a) **2** (c = 5 × 10^{-7} mol dm^{-3}; λ_{exc}= 350 nm) with $Cd(ClO_4)_2 \times H_2O$ (c = 5 × 10^{-4} mol dm^{-3}); b) agreement between experimental and calculated data at 360 nm; c) percentage of chromophore species in solution in dependence to c(metal cation). Titration data are processed by using the Specfit program. Done in water; r = [$Cd(ClO_4)_2 \times H_2O$] / [compound].

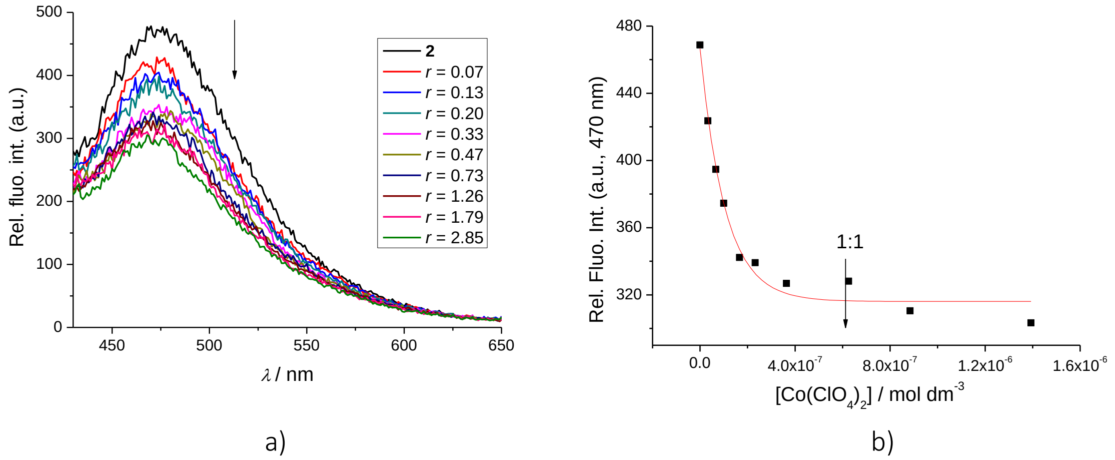

Figure S34. Fluorimetric titration of: a) **2** ($c = 5 \times 10^{-7}$ mol dm^{-3}; λ_{exc}= 350 nm) with $Co(ClO_4)_2 \times 6\ H_2O$ ($c = 5 \times 10^{-5}$ mol dm^{-3}) and b) agreement between experimental and calculated data at 470 nm. The end of titration at ratio [dye] / [metal cation] = 1:1 strongly supports log $K > 8$.

4.4. Titrations of pyrene derivatives **2** and **3** with TMACl

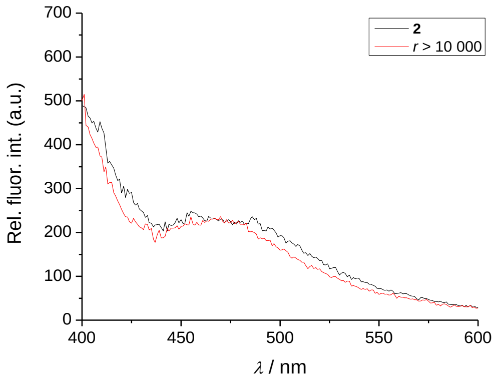

Figure S35. Dependence of fluorescence of **2** ($c = 1 \times 10^{-6}$ mol dm^{-3}; λ_{exc} = 350 nm) with TMACl (tetramethylammonium chloride, c = 0.1 mol dm^{-3}). Done in water; r = [compound] / [TMACl], λ_{max} = 475 nm.

5. Fluorescence lifetime measurements

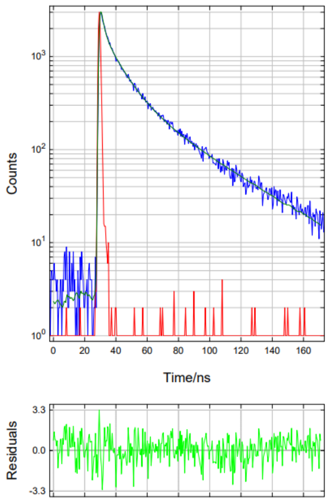

Parameter	Value	Std. Dev.	Rel %
τ1	1.673E-009 s	1.028E-010 s	
τ2	1.072E-008 s	4.191E-010 s	
τ3	3.894E-008 s	9.226E-010 s	
Shift	2.054E-011 s	8.203E-012 s	
B1	0.2735	0.0091	10.83
B2	0.1660	0.0048	42.13
B3	0.0510	0.0028	47.04
A	0.8835		
χ^2	1.1472		

a)

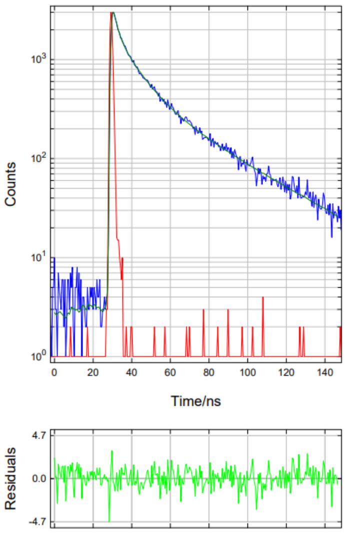

Parameter	Value	Std. Dev.	Rel %
τ1	1.883E-009 s	1.894E-010 s	
τ2	1.013E-008 s	5.684E-010 s	
τ3	3.646E-008 s	1.018E-009 s	
Shift	-1.058E-011 s	2.489E-011 s	
B1	0.2493	0.0131	10.94
B2	0.1619	0.0069	38.22
B3	0.0598	0.0038	50.84
A	1.2550		
χ^2	1.1804		

b)

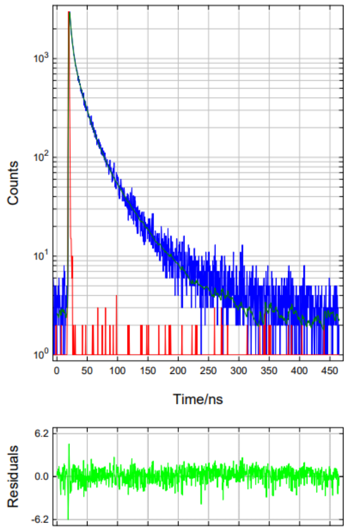

Parameter	Value	Std. Dev.	Rel %
τ1	2.861E-009 s	1.387E-010 s	
τ2	1.499E-008 s	5.600E-010 s	
τ3	4.700E-008 s	1.467E-009 s	
Shift	-4.644E-011 s	1.723E-011 s	
B1	0.2782	0.0072	20.14
B2	0.1309	0.0042	49.65
B3	0.0254	0.0021	30.21
A	0.9143		
χ^2	1.2080		

c)

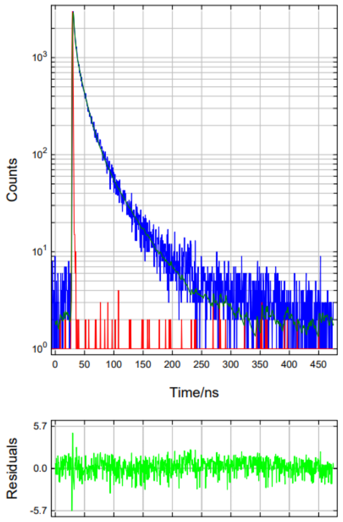

Parameter	Value	Std. Dev.	Rel %
τ_1	2.086E-009 s	1.169E-010 s	
τ_2	1.258E-008 s	4.103E-010 s	
τ_3	4.218E-008 s	1.108E-009 s	
Shift	-1.326E-010 s	1.683E-011 s	
B1	0.3000	0.0096	17.65
B2	0.1434	0.0040	50.91
B3	0.0264	0.0018	31.44
A	0.6049		
χ^2	1.2078		

d)

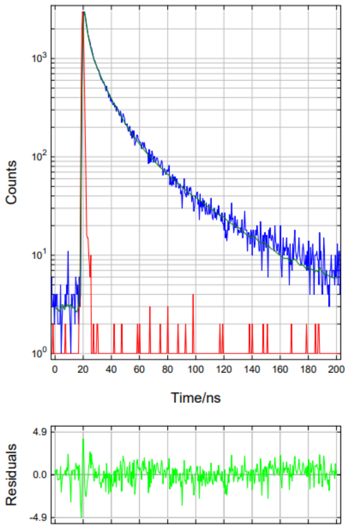

Parameter	Value	Std. Dev.	Rel %
τ_1	1.709E-009 s	1.019E-010 s	
τ_2	1.068E-008 s	3.644E-010 s	
τ_3	3.873E-008 s	1.210E-009 s	
Shift	-1.941E-010 s	1.624E-011 s	
B1	0.3244	0.0114	16.80
B2	0.1542	0.0045	49.92
B3	0.0284	0.0020	33.28
A	1.4402		
χ^2	1.2109		

e)

Figure S36. Decay profile of: a) ligand **2** (mono-pyrene conjugate) ($c = 5 \times 10^{-6}$ mol dm^{-3}); b) **2**-Cu^{2+} complex at r = 100; c) **2**-Cu^{2+} complex at r = 10; d) **2**-Cu^{2+} complex at r = 1; e) **2**-Cu^{2+} complex at r = 0.1. Done in Milli Q water, r = [**2**] / [$Cu(ClO_4)_2 \times 6H_2O$]. λ_{em} = 475 nm.

Table S2. Fluorescence lifetime data of **2**, and **2**-Cu^{2+} complex in Milli Q water. Samples were excited at 340 nm (EPLED340 (340.1 nm is the correct wavelength for this EPLED)). Emission was monitored at 475 nm. Fitted to 3 species.

	^{a}r	$^{b}\lambda_{exc}$ [nm]	λ_{em} [nm]	$^{a}\tau$ [ns]	χ^2
**2**	-	340	475	1.67 (10.83 %)<br>10.72 (42.13 %)<br>38.94 (47.04 %)	1.1472
**2**-Cu^{2+}	100	340	475	1.88 (10.94 %)<br>10.13 (38.22 %)<br>36.46 (50.84 %)	1.1804
**2**-Cu^{2+}	10	340	475	2.86 (20.14 %)<br>14.99 (49.65 %)<br>47.00 (30.21 %)	1.208
**2**-Cu^{2+}	1	340	475	2.086 (17.65 %)<br>12.58 (50.91 %)<br>42.18 (31.44 %)	1.2078
**2**-Cu^{2+}	0.1	340	475	1.71 (16.8 %)<br>10.68 (49.92 %)<br>38.73 (33.28 %)	1.0769

[a] r = [**2**] / [$Cu(ClO4)_2 \times 6H_2O$]

[b] Samples were excited by pulsing diode EPLED340 at 340.1 nm. Emission was monitored at 475 nm. Aqueous solutions were purged by argon.

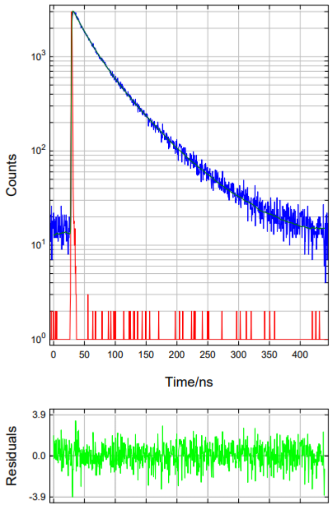

Parameter	Value	Std. Dev.	Rel %
τ_1	2.343E-008 s	1.977E-008 s	
τ_2	2.557E-008 s	1.267E-008 s	
τ_3	6.171E-008 s	1.795E-009 s	
Shift	6.014E-011 s	8.928E-012 s	
B1	0.0675	0.6785	11.14
B2	0.1248	0.6787	22.50
B3	0.1525	0.0161	66.36
A	8.1894		
χ^2	1.0286		

a)

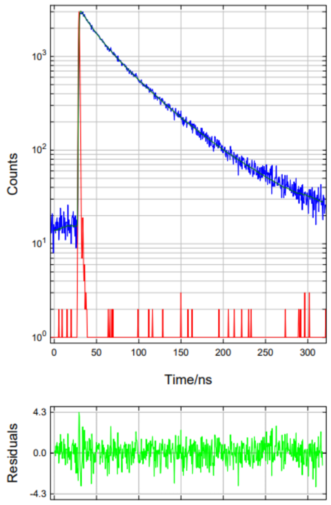

Parameter	Value	Std. Dev.	Rel %
τ_1	1.399E-008 s	3.401E-009 s	
τ_2	3.448E-008 s	9.943E-009 s	
τ_3	6.373E-008 s	6.108E-009 s	
Shift	4.500E-011 s	8.547E-012 s	
B1	0.0905	0.0433	8.95
B2	0.1510	0.0233	36.78
B3	0.1205	0.0545	54.27
A	10.1937		
χ^2	1.1237		

b)

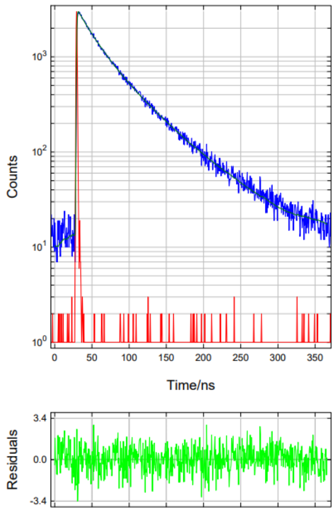

Parameter	Value	Std. Dev.	Rel %
τ_1	2.933E-009 s	3.385E-008 s	
τ_2	2.240E-008 s	7.310E-010 s	
τ_3	5.854E-008 s	8.599E-010 s	
Shift	-2.191E-010 s	3.970E-011 s	
B1	0.0061	0.0318	0.13
B2	0.1866	0.0053	31.28
B3	0.1566	0.0063	68.59
A	7.3866		
χ^2	1.0888		

c)

Figure S37. Decay profile of: a) ligand **3** (bis-pyrene conjugate) (c = 5 × 10^{-6} mol dm^{-3}); b) **3**-Cu^{2+} complex at r = 10; c) **3**-Cu^{2+} complex at r = 0.1. Done in Milli Q water, r = [**2**] / [$Cu(ClO_4)_2$ × $6H_2O$]. λ_{em} = 475 nm.

Table S3. Fluorescence lifetime data of **3**, and **3**-Cu^{2+} complex in Milli Q water. Samples were excited at 340 nm (EPLED340 (340.1 nm is the correct wavelength for this EPLED)). Emission was monitored at 475 nm.

	[a]r	[b]λ_{exc} [nm]	λ_{em} [nm]	[a]τ [ns]	χ^2
**3**	-	340	475	23.43 (11.14 %)<br>25.57 (22.50 %)<br>61.71 (66.36 %)	1.0286
**3**-Cu^{2+}	10	340	475	13.99 (8.95 %)<br>34.48 (36.78 %)<br>63.73 (54.27 %)	1.1237
**3**-Cu^{2+}	0.1	340	475	2.93 (0.13 %)<br>22.40 (31.28 %)<br>58.54 (68.59 %)	1.0888

[a] r = [**3**] / [$Cu(ClO4)_2 \times 6H_2O$]

[b] Samples were excited by pulsing diode EPLED340 at 340.1 nm. Emission was monitored at 475 nm. Aqueous solutions were purged by argon.

6. Computational simulations protocols

Structures of mono- and bis-pyrene derivatives and their Zn(II)-metal complexes were optimized in Gaussian 16 program package, Revision C.01 [1]. Initially, zinc cation was placed between imidazole nitrogen atoms with distances of approximatelly 2.2 Å and pyrene arms were in extended conformation attached to calixarene lower rim. Geometries were optimized in water on B3LYP/6-31G(d,p) level of theory [2,3] and for zinc atom the pseudopotential LANL2DZ basis set for transition metals was used [4]. Water was described implicitly by conducting the PCM (Polarizable Continuum Model) [5] with the DFT-B3LYP method. Minima on the potential energy surfaces were confirmed by calculating the harmonic vibrational frequencies.

In order to perform the classical molecular dynamics (MD) simulations of pyrene-conjugates and their metal complexes, the unknown force field parameters for zinc-ligand coordination were obtained by using the VFFDT (Visual Force Field Derivation Toolkit) software. This program has recently shown very good performance for evaluating bonded (bond lengths and angles) and nonbonded (van der Waals, electrostatic) force field parameters for metal-molecular systems for MD simulations [6]. For that purpose, the harmonic vibrational frequencies necessary for angle and bond parameters were calculated at B3LYP/6-31G(d,p) level of theory [2,3] and atomic partial charges according to RESP (Restrained Electrostatic Potential Charges) procedure (HF/6-31G(d)); LANL2DZ for zinc in both cases [7]. Calixarenes were described using the GAFF2 (Generalized Force Field 2) parameters that are suggested for organic ligands [8]. The systems were simulated in TIP3P water cubical box (dimension 65 Å) [9], consisting of water molecules treated explicitly and two Cl^{-} ions added to metal-complex complexes to neutralize the system.

Each simulation was performed in four steps: minimization, heating, equilibration, and final production (MD simulation) of the system. Geometry was optimized in the first 5000 steps with the steepest descent method and the rest of the steps with the conjugate gradient method without any constraints. After the minimization, the system was heated for 5 ps from 0-100 K at constant volume (NVT ensemble, Langevin thermostat) [10] and 100 ps from 100-303 K at constant pressure (p = 1 atm, NpT ensemble, Monte Carlo barostat, anisotropic pressure coupling, pressure relaxation time 2 ps). 100 ns of equilibration step was performed at constant pressure (same conditions) while the production step was obtained for 100 ns. Three-dimensional periodic boundary conditions were used throughout. Time step for all simulations was 2 fs and the coordinates were saved. The cutoff for short range Coulomb interactions and van der Waals interactions was set to 12 Å, with the switching function for the latter turned on after 10 Å. Long range interactions were handled using particle mesh Ewald procedure (PME) [11]. Bonds involving hydrogen were constrained with SHAKE [12]. Simulations were performed in Amber22 program package [13] while the figures were prepared in Pymol [14].

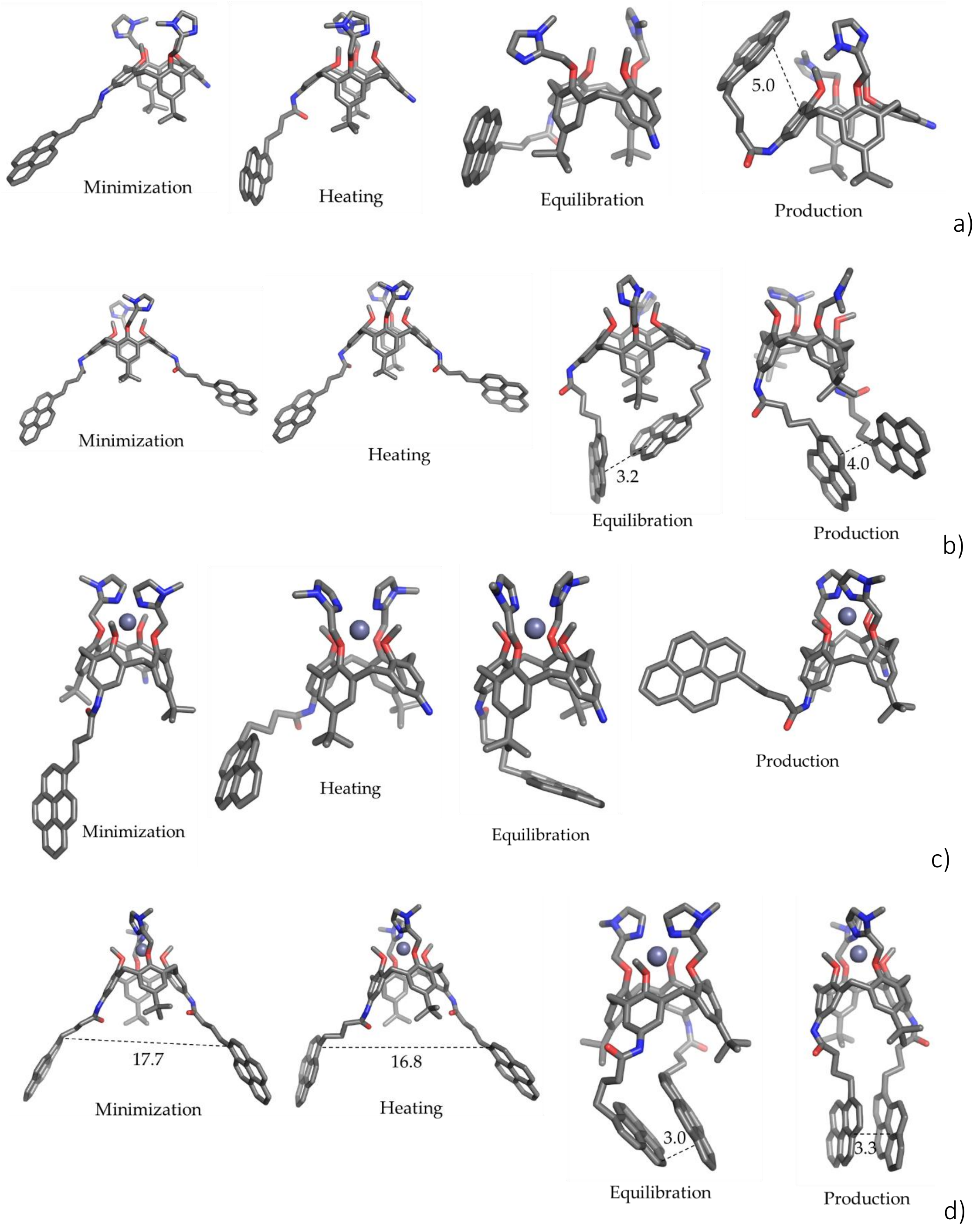

Figure S38. Structural rearrangements of mono-and bis-pyrene derivatives (a) and b)) and their Zn^{2+} complexes (c) and d)) during the different steps of MD simulations: minimization, heating, equilibration and production step (100 ns). Shortest distances are given in Å.

7. References

1. Frisch, M.J.; Trucks, G.W.; Schlegel, H.B.; Scuseria, G.E.; Robb, M.A.; Cheeseman, J.R.; Scalmani, G.; Barone, V.; Petersson, G.A.; Nakatsuji, H.; et al. Gaussian 16, Revision C.01; Gaussian, Inc.: Wallingford, CT, USA, 2016.
2. Raghavachari, K. Perspective on "Density functional thermochemistry. III. The role of exact exchange" Becke AD (1993) J Chem Phys 98: 5648–52. Theor. Chem. Acc. 2000, 103, 361–363. https://doi.org/10.1007/s002149900065.
3. Krishnan, R.; Binkley, J.S.; Seeger, R.; Pople, J.A. Self-consistent molecular orbital methods. XX. A basis set for correlated wave functions. Chem. Phys. 1980, 72, 650–654. https://doi.org/10.1063/1.438955.
4. Chiodo, S.; Russo, N.; Sicilia, E. LANL2DZ basis sets recontracted in the framework of density functional theory. J. Chem. Phys. 2006, 125, 104107. https://doi.org/10.1063/1.2345197.
5. Mennucci, B. Polarizable continuum model. WIREs Comput. Mol. Sci. 2012, 2, 386–404. https://doi.org/10.1002/wcms.1086.
6. Zheng, S.; Tang, Q.; He, J.; Du, S.; Xu, S.; Wang, C.; Xu, Y.; Lin, F. VFFDT: A new software for preparing AMBER force field parameters for metal-containing molecular systems. J. Chem. Inf. 2016, 56, 811–818. https://doi.org/10.1021/acs.jcim.5b00687.
7. Bayly, C.I.; Cieplak, P.; Cornell, W.; Kollman, P.A. A well-behaved electrostatic potential based method using charge restraints for deriving atomic charges: the RESP model. J. Phys. Chem. 1993, 97, 10269–10280. https://doi.org/10.1021/j100142a004.
8. Vassetti, D.; Paglia, M.; Procacci, P. Assessment of GAFF2 and OPLS-AA General Force Fields in Combination with the Water Models TIP3P, SPCE, and OPC3 for the Solvation Free Energy of Druglike Organic Molecules. J. Chem. Theory Comput. 2019, 15, 1983–1995. https://doi.org/10.1021/acs.jctc.8b01039.
9. Price, D.J.; Brooks, C.L. A modified TIP3P water potential for simulation with Ewald summation. J. Chem. Phys. 2004, 121, 10096–100103. https://doi.org/10.1063/1.1808117.
10. Goga, N.; Rzepiela, A.J.; de Vries, A.H.; Marrink, S.J.; Berendsen, H.J.C. Efficient Algorithms for Langevin and DPD Dynamics. J. Chem. Theory Comput. 2012, 8, 3637–3649. https://doi.org/10.1021/ct3000876.
11. Darden, T.; York, D.; Pedersen, L. Particle mesh Ewald: An N· log (N) method for Ewald sums in large systems. J. Chem. Phys. 1993, 98, 10089–10092. https://doi.org/10.1063/1.464397.
12. Kräutler, V.; Van Gunsteren, W.F.; Hünenberger, P.H. A fast SHAKE algorithm to solve distance constraint equations for small molecules in molecular dynamics simulations. J. Comput. Chem. 2001, 22, 501–508. https://doi.org/10.1002/1096-987X(20010415)22:5%3C501::AID-JCC1021%3E3.0.CO;2-V.
13. Case, D.A.; Aktulga, H.M.; Belfon, K.; Ben-Shalom, I.Y.; Berryman, J.T.; Brozell, S.R.; Cerutti, D.S.; Cheatham, T.E., III; Cisneros, G.A.; Cruzeiro, V.W.D.; et al. Amber 2022; University of California: San Francisco, CA, USA, 2022.
14. Schrodinger, L. The PyMOL Molecular Graphics System. Version 1.8; Schrödinger: New York, NY, USA, 2015.
